# Optimal Sensor Placement for Bridge Structural Health Monitoring: A Systematic Review with Bibliometric Analysis and Methodological Quality Assessment

**DOI:** 10.3390/s26206661

**Published:** 2026-10-21

**Authors:** Gastón Luis Rodríguez Wlack, Nicolás Fuentes, Álvaro Peña Fritz, Broderick Crawford, Ricardo Soto

**Affiliations:** 1 Pontificia Universidad Católica de Valparaíso, Avenida Brasil 2147, Valparaíso 2362807, Chile; alvaro.pena@pucv.cl; 2 Escuela de Ingeniería Informática, Pontificia Universidad Católica de Valparaíso, Avenida Brasil 2241, Valparaíso 2362807, Chile; nicolas.fuentes.e@mail.pucv.cl (N.F.); broderick.crawford@pucv.cl (B.C.); ricardo.soto@pucv.cl (R.S.)

**Keywords:** sensor placement, structural health monitoring, bridge monitoring, metaheuristic optimization, optimal sensor placement, damage detection, vibration

## Abstract

Optimal Sensor Placement (OSP) is a key step in the design of Structural Health Monitoring (SHM) systems for bridges and requires combining civil engineering constraints with computational optimization. This systematic review, conducted following PRISMA 2020 guidelines, analyzes 49 studies through bibliometric analysis, a synthesis of optimization strategies, sensor modalities and research motivations, and a tailored Quality Assessment rubric. Accordingly, its quantitative results describe publication patterns, coded categories and methodological quality scores rather than bridge-specific dynamic parameters. Accelerometers are the most frequently used sensor modality, and improving data quality is the most frequently reported motivation (45 of 49 studies), whereas cost reduction, communication efficiency and energy efficiency appear in 11 studies each. Only 15 studies included experimental or field validation on physical bridges, and robustness against uncertainty was the weakest quality dimension, revealing a persistent simulation-to-reality gap. To organize these findings, the Sensor Placement Optimization Maturity Levels (SPO-ML) typology and a Unified Operational Decision Framework are proposed as literature-informed, preliminary guidance relating algorithm selection to structural complexity and network constraints. Future OSP research should prioritize field validation, robustness under uncertainty, and reproducible reporting of bridge and instrumentation characteristics.

## 1. Introduction

The optimal placement of sensors on bridges is a key aspect of Structural Health Monitoring (SHM), aiming to ensure the safety and longevity of critical infrastructure. As the demand for reliable and efficient monitoring systems increases [1], the challenge of effective sensor positioning becomes crucial. Given the high costs associated with sensors and their installation, a well-planned sensor placement strategy is essential to maximize data quality while minimizing costs [1]. Recent advances in metaheuristic optimization algorithms have shown significant potential for improving sensor placement strategies, enabling greater accuracy and reliability in monitoring dynamic environments [2].

Prominent examples of bridges employing SHM systems include the Golden Gate Bridge in the United States, the Viaduct de Millau in France, and the Hangzhou Bay Bridge in China. These structures utilize advanced sensor networks to monitor their structural integrity in real-time, ensuring safety and enabling predictive maintenance. Such examples highlight the importance of optimizing sensor placement to maximize the effectiveness of these monitoring systems.

There are already well-established and functional methods for Optimal Sensor Placement (OSP) that balance the acquisition of relevant information with the costs associated with sensor installation. Among these methods, the most notable are Modal Kinetic Energy (MKE), Effective Independence (EFI), Information Entropy Index (IEI), and MinMAC. These techniques have been successfully applied to real-world structures, such as pedestrian bridges, using modal analyses derived from finite element models to guide sensor placement [3].

One of the most widely adopted approaches is the EFI method, which seeks to maximize the linear independence of the captured modal shapes, allowing the extraction of the most relevant information with relatively low computational cost. Conversely, the MKE method selects locations based on the modal kinetic energy contribution of each point, while the IEI evaluates positions in terms of information entropy, and MinMAC focuses on minimizing the correlation between measured modal shapes and the model. These methods are commonly assessed using metrics such as the determinant of the Fisher Information Matrix (DET), the Information Entropy Index (IEI), and the Root Mean Square (RMS) of the off-diagonal entries of the Modal Assurance Criterion (MAC) matrix.

Recent studies have demonstrated that the EFI method, applied to structures such as the Streicker pedestrian bridge, can estimate optimal sensor positions while providing significant structural information, even with configurations involving a limited number of sensors. This type of approach highlights that, despite budgetary or accessibility constraints, the methods currently available are effective in meeting SHM objectives and optimizing the monitoring of dynamic structures.

One of the major challenges in optimal sensor placement lies in the interdisciplinary gap between civil engineering and optimization sciences. Advanced optimization methods, such as genetic algorithms and deep reinforcement learning, have demonstrated substantial potential for enhancing sensor placement. However, their practical implementation in civil engineering is limited due to civil engineers’ lack of familiarity with these techniques, which are often considered outside their domain of expertise [4]. Similarly, optimization researchers often lack a deep understanding of the specific needs and operational constraints of civil structures [5]. Conventional sensor placement strategies frequently rely on modal information obtained from structural models or experimental modal analysis. Figure 1 illustrates how global mode shapes reveal spatial differences in structural response that can guide the selection of informative sensor locations. This modal information underpins established OSP criteria such as EFI, MKE, and MinMAC, although these approaches may not explicitly account for additional operational constraints such as communication, energy consumption, or sensor heterogeneity [4]. Bridging this interdisciplinary disconnect requires not only the development of more robust and accessible methods but also a concerted effort to foster collaboration between these fields.

The motivation for this systematic review stems from the need to assess the current state of research on sensor placement optimization in the context of SHM for bridges. Effective sensor placement not only facilitates the monitoring of structural integrity but also enables the prediction of potential failures before they occur, thereby enhancing operational safety [7]. By analyzing various methodologies and optimization techniques employed in the literature, this review aims to provide a comprehensive understanding of existing approaches and their effectiveness in real-world applications.

Existing reviews have examined different aspects of OSP and SHM, including wireless sensor placement, algorithmic developments, and structural monitoring applications [8,9,10]. However, these contributions have generally examined algorithms, sensor technologies, or monitoring applications separately. A systematic synthesis relating to algorithmic strategy, structural complexity, sensor modality, operational motivation, methodological quality, and practical engineering applicability remains limited. Consequently, a consolidated framework translating these dimensions into engineering-oriented selection criteria for bridge SHM remains limited.

To address the challenges identified above, this review seeks to answer three critical research questions, focusing on strategies, sensor characteristics, and the benefits of optimal sensor placement for SHM:

- RQ1: What sensor placement optimization strategies have been investigated for bridge SHM, and how have they been evaluated?
- RQ2: What types of sensors are commonly used in bridge structural monitoring, and what are their main characteristics?
- RQ3: What are the primary motivations for applying optimal sensor placement in bridge monitoring, and what benefits have been empirically or quantitatively demonstrated?

Additionally, the review provides a reference framework for determining when it is necessary to employ an intelligent sensor placement strategy, considering the specific characteristics of each infrastructure. It aims to offer guidance for selecting the most appropriate algorithm, assisting professionals in making informed decisions about the use of optimization algorithms in structural monitoring without requiring an in-depth understanding of the mathematical details of each technique.

It should be noted that the quantitative component of this review is bibliometric and methodological: it comprises publication counts, the frequency of coded algorithmic, sensor and motivation categories, and quality assessment scores. Bridge-specific dynamic parameters, such as natural frequencies (e.g., Figure 1), damping ratios, recorded accelerations or installed sensor coordinates, are not systematically compiled, since they are reported heterogeneously across the reviewed studies and are frequently derived from numerical models only. Accordingly, the proposed framework is intended as preliminary guidance and does not replace a bridge-specific design of the monitoring system.

## 2. Materials and Methods

### 2.1. Methodology

The research methodology established for this systematic review aims to address the central research questions (RQs) previously outlined, which focus on the optimization of sensor placement in bridge SHM. To effectively tackle these questions, three key stages were defined.

The first stage involved defining key concepts related to SHM systems and optimization strategies. These definitions were essential not only for guiding the selection of articles to be reviewed but also for providing a foundation for the development of a structured search strategy. By clearly identifying the relevant terms and concepts, this stage ensured that the search process was systematic, targeted, and aligned with the objectives of the review. Within this framework, the timeframe of 2010 to 2025 was established as a technical milestone, marking the beginning of the widespread integration of metaheuristic optimization and the transition toward autonomous wireless sensor networks in civil infrastructure monitoring.

In the second stage, a detailed analysis was conducted on the algorithms used for sensor placement, as well as the types of sensors most employed, performance metrics, and the benefits associated with their use in structural health assessment of bridges. This stage also included an analysis of how each algorithm responds to the motivations identified in the first stage.

The third stage involved a systematic evaluation of the quality and risk of bias of the included studies. By applying a multidimensional scoring framework, the evidence was hierarchically categorized based on its validation rigor, structural complexity, reproducibility, and robustness against uncertainty. This final stage ensured that the synthesis of results and the proposed operational decision framework were founded on research demonstrating high technical fidelity and applicability in real-world environments.

Figure 2 presents a conceptual representation of the proposed research methodology, where the stages, tools, activities, and corresponding outcomes can be identified. Through the review and subsequent quality assessment of the selected articles, the most relevant aspects regarding sensor placement optimization strategies, the algorithms used, and the performance evaluation metrics applied in the studies were established. This allowed the categorizing of the contributions of the articles in a comparative manner, interpreting their similarities and differences, and providing a comprehensive understanding of the approaches and methodologies employed in the SHM field for bridges.

### 2.2. Search Strategy

This review follows the PRISMA 2020 guidelines for systematic reviews. The review protocol was not prospectively registered. Web of Science (WoS) and IEEE Xplore were selected as the primary databases for the formal systematic search. During the preliminary scoping stage, exploratory searches were also conducted in Scopus and the ASCE Library, which showed substantial overlap with the relevant literature subsequently retrieved through WoS and IEEE Xplore. Based on this preliminary assessment, the formal search was restricted to these two databases to reduce database redundancy while maintaining complementary coverage of the interdisciplinary nature of OSP research. WoS provided broad multidisciplinary coverage, including structural and civil engineering research, whereas IEEE Xplore complemented this source with literature related to computational optimization, wireless sensor networks, and intelligent monitoring systems. Google Scholar was used only as a supplementary source for targeted documentation and was not included in the formal systematic screening process.

For both WoS and IEEE Xplore, advanced queries were defined and tailored to the syntax of each database. These queries were based on keywords and phrases closely related to the research topic, and logic was employed to exclude irrelevant terms at this stage. The database-specific search strings are presented in Table 1.

Following the retrieval of articles, a systematic filtering process was carried out to ensure the relevance and quality of the selected studies. This process was guided by predefined inclusion and exclusion criteria, which are comprehensively outlined in Table 2. These criteria were designed to focus on studies that align with the objectives of this review while excluding those that do not meet the required standards or level of relevance. To provide a clear overview of the filtering methodology, Figure 3 illustrates the entire process, detailing each stage from the initial search to the final selection of articles. This approach ensures transparency and reproducibility in the article selection process.

The final selection of articles is summarized in Table 3. These 49 studies met all inclusion criteria and provided the foundation for the analysis conducted in this systematic review.

The initial search identification across the selected databases yielded a total of 281 records (239 from WoS and 42 from IEEE Xplore). After the removal of 2 duplicate records, 279 articles entered the screening phase based on title and abstract analysis. In this stage, 214 records were excluded for not being specifically focused on bridge structures (EC1). Subsequently, 65 reports were sought for full-text retrieval and assessed for eligibility. During this final filtering, 16 reports were excluded based on the following predefined criteria: (i) 6 studies showed insufficient technical quality or lacked a rigorous peer-review process (EC2), and (ii) 10 studies were excluded for lacking a formal structural validation phase, such as high-fidelity numerical simulations or experimental testing specifically applied to bridge structures (EC3). This systematic process resulted in a final selection of 49 studies included in qualitative and quantitative synthesis.

### 2.3. Quality Assessment and Risk of Bias Scoring

To comply with the PRISMA 2020 guidelines and ensure the robustness of the systematic review, a quantitative Quality Assessment (QA) and Risk of Bias (RoB) framework was specifically designed for the interdisciplinary domain of Optimal Sensor Placement (OSP) in Structural Health Monitoring (SHM). Traditional medical-focused RoB tools are inadequate for engineering algorithmic reviews; therefore, adapting QA criteria to evaluate methodological rigor, experimental design, and algorithm reproducibility is recognized as a necessary practice in computational and SHM systematic reviews [51,52].

Consequently, a tailored scoring system was implemented to evaluate the level of evidence of the 49 included studies, penalizing methodological flaws that hinder real-world structural validation [53].

For the Structural Complexity and Scalability dimension, the DOF ranges were defined as heuristic order-of-magnitude categories to enable consistent comparison of structural models across the reviewed studies. The boundaries at 1000 and 10,000 DOFs distinguish reduced models involving hundreds of DOFs, intermediate models involving thousands of DOFs, and large-scale models reaching tens of thousands of DOFs. These ranges are used solely as comparative categories within the review and should not be interpreted as validated engineering thresholds.

The proposed QA framework evaluates each study across four technical dimensions. A score ranging from 0 to 3 is assigned to each category, yielding a maximum possible score of 12 points (S_total). The evaluation criteria for each dimension are detailed in Table 4.

DOF ranges represent indicative order-of-magnitude categories adopted for comparative assessment and are not validated engineering thresholds.

Based on the cumulative score (S_total), the included studies are hierarchically categorized into three levels of evidence to evaluate the risk of bias:

- High Quality (Low Risk of Bias): 9 to 12 points.
- Moderate Quality (Moderate Risk of Bias): 5 to 8 points.
- Low Quality (High Risk of Bias): 0 to 4 points.

This stratification allows the subsequent synthesis of strategies, algorithms, and sensor characteristics to place greater interpretive emphasis on methodologies demonstrating higher technical maturity and applicability to real-world civil infrastructure projects. The detailed scoring for each of the 49 reviewed articles is consolidated in Table 5.

### 2.4. Data Extraction and Coding Protocol

To ensure the analytical traceability and reproducibility of this systematic review, a standardized data extraction protocol was implemented, directly aligned with the tools defined in Stage 2 of the research methodology (Figure 2). Upon completion of the study selection phase, the full texts of the 49 eligible studies were independently reviewed and coded by two researchers. A structured data extraction matrix was developed to systematically capture relevant information and mitigate subjective interpretation.

The coding framework was specifically designed to address the central Research Questions (RQs) and encompassed three primary domains: (i) *Bibliometric Data* (e.g., authors, publication year, country of origin); (ii) *Methodological Data* (e.g., optimization algorithm typology to address RQ1, objective function mathematical formulation, and structural complexity); and (iii) *Technical Implementation* (e.g., sensor modalities utilized to address RQ2, and primary research motivations to address RQ3).

To validate the extraction process and ensure inter-coder reliability, a blind coding pilot was conducted prior to the full extraction. The two reviewers independently coded a random subset of 15 articles (approximately 30% of the sample). The inter-rater agreement was statistically evaluated using Cohen’s Kappa coefficient. The analysis yielded a value of 0.86, indicating strong agreement between the coders. Any discrepancies identified during the coding of the full dataset were resolved through iterative consensus discussions or, when necessary, by consulting a third senior researcher. This rigorous coding protocol ensures that the subsequent categorization and data synthesis are rooted in a verifiable, reproducible, and unbiased analytical framework.

### 2.5. Analysis Categories

Building upon the robust dataset generated through the coding protocol, three key criteria were defined to guide the systematic synthesis. In the field of structural health monitoring, optimizing sensor placement is crucial for ensuring accurate data collection, cost efficiency, and effective structural analysis. These criteria serve as the foundation for the subsequent analysis, map directly to the extracted data domains, and are summarized below for clarity.

The first criterion addresses the algorithms used for sensor placement optimization. These algorithms play a pivotal role in determining optimal sensor configurations by balancing multiple objectives, such as maximizing data quality, reducing redundancy, and meeting practical constraints. Techniques such as genetic algorithms, deep learning, and other metaheuristic approaches have shown significant potential in this domain. Figure 4 shows the criteria and subcategories corresponding to the research question related to strategies for the optimal positioning of sensors.

The second criterion examines the types of sensors commonly deployed in SHM and their specific operational characteristics. This category encompasses diverse instrumentation, such as accelerometers, strain gauges, and thermal sensors, each providing unique structural response characteristics. Sensor selection is inherently dictated by the structural behavior under evaluation and the required measurement precision. Figure 5 illustrates the criterion and subcategories addressing research questions regarding sensor typology.

The third criterion investigates the underlying motivations for optimizing sensor placement, reflecting the operational and economic objectives driving the research. These motivations range from cost reduction, achieved by minimizing sensor count without degrading measurement accuracy, to enhancing data reliability through strategic positioning. Furthermore, optimization aims to improve communication efficiency within Wireless Sensor Networks (WSNs) and maximize the signal-to-noise ratio. Figure 6 presents the criterion and subcategories related to optimization motivations.

Collectively, these three criteria establish a structured analytical framework. The detailed descriptions of the analysis categories and subcategories, condensing algorithmic approaches, sensor typologies, and optimization motivations, are presented in Table 6. This systematic categorization facilitates a comprehensive evaluation of the state-of-the-art methodologies applied to bridge SHM, ensuring that the subsequent discussion is grounded in the high-fidelity evidence identified during the quality assessment phase.

The analytical categories were not treated as mutually exclusive. A study could therefore be assigned to more than one algorithmic, sensor, or motivation category when multiple approaches were explicitly addressed within the same work. Consequently, aggregated category counts may exceed the total number of included studies.

## 3. Results

This section aims to provide a comprehensive analysis of the articles included in the systematic literature review, following an approach that moves from a general perspective to specific details. It systematically addresses the research questions posed, categorizing the information to facilitate the understanding of the most significant findings.

First, a global synthesis is conducted to identify common patterns and trends in the strategies and approaches employed in the reviewed studies. Subsequently, the technical and methodological aspects of the articles are examined in detail, highlighting the most relevant contributions to the optimal placement of sensors on bridges for structural health monitoring.

Finally, a summary is provided to underscore the key points of this investigation, synthesizing the most relevant results and suggesting potential directions for future research in this field. This approach not only provides comprehensive answers to the research questions but also offers a critical and comparative perspective that enhances the state of the art in this domain.

### 3.1. Key Characteristics of the Reviewed Articles

Following the methodological framework established in Section 2.3, the 49 included studies were quantitatively evaluated across four technical dimensions: Validation Method, Structural Complexity, Reproducibility, and Robustness against Uncertainty. This scoring process is critical for identifying the risk of bias and ensuring that the subsequent technical analysis is primarily driven by highly mature and applicable research.

The detailed scoring for each article is presented in Table 5, organized by evidence tier (High, Moderate, and Low Quality) for clarity. The distribution of the cumulative scores (S_total) reveals the current technological maturity of Optimal Sensor Placement (OSP) algorithms in civil engineering. Studies classified as High Quality demonstrate robust field validations or high-fidelity numerical models applied to large-scale structures, providing the most reliable evidence for the proposed decision frameworks. Conversely, studies with Moderate- or Low-Quality scores generally exhibit limitations in scalability or rely heavily on theoretical, noise-free 2D models, highlighting the persistent simulation-to-reality gap in the field.

**Table 5:** Quality assessment and risk of bias scoring for the included studies.

Study (Reference)	Validation Method (0–3)	Structural Complexity (0–3)	Reproducibility (0–3)	Robustness (0–3)	Total Score (S_total)	Evidence Level
**High Quality 9 to 12 points**						
B. Li et al. [6]	3	2	3	3	**11**	High
Febrianto et al. [17]	3	3	2	3	**11**	High
Liu et al. [31]	3	3	2	3	**11**	High
Xu et al. [39]	3	3	2	3	**11**	High
Feng and Jia [18]	2	3	2	3	**10**	High
Guo et al. [24]	2	3	2	3	**10**	High
Zhang et al. [43]	3	3	2	2	**10**	High
Cherid et al. [13]	2	3	2	2	**9**	High
Pachón et al. [33]	3	3	2	1	**9**	High
**Moderate Quality 5 to 8 points**						
D. Shan et al. [14]	2	3	2	1	**8**	Moderate
D. Xiao et al. [15]	2	2	2	2	**8**	Moderate
Flynn and Todd [20]	3	1	1	3	**8**	Moderate
Gao et al. [23]	2	2	2	2	**8**	Moderate
J. C. Avendano et al. [26]	2	3	2	1	**8**	Moderate
Martin and Droguett [1]	3	2	2	1	**8**	Moderate
S et al. [34]	3	0	2	3	**8**	Moderate
Y. Zhang et al. [40]	2	1	2	3	**8**	Moderate
Yang et al. [41]	3	1	2	2	**8**	Moderate
Chen et al. [8]	3	1	1	2	**7**	Moderate
Dong et al. [16]	3	1	2	1	**7**	Moderate
Feng and Jia [19]	2	3	2	0	**7**	Moderate
G. Li et al. [21]	2	3	2	0	**7**	Moderate
G. W. van der Linden et al. [22]	1	1	2	3	**7**	Moderate
He et al. [25]	2	2	2	1	**7**	Moderate
J. Yang and Z. Peng [27]	2	2	2	1	**7**	Moderate
Li et al. [28]	3	1	2	1	**7**	Moderate
Meng et al. [5]	2	2	2	1	**7**	Moderate
van der Linden et al. [37]	1	1	2	3	**7**	Moderate
Wu et al. [4]	3	1	2	1	**7**	Moderate
Zhou and Wu [50]	2	1	2	2	**7**	Moderate
Zhou et al. [2]	2	2	2	1	**7**	Moderate
Zhou et al. [46]	2	2	2	1	**7**	Moderate
Chai et al. [12]	1	1	2	2	**6**	Moderate
Karakostas et al. [9]	2	2	1	1	**6**	Moderate
Laory et al. [7]	1	1	2	2	**6**	Moderate
Li et al. [29]	2	2	2	0	**6**	Moderate
Li et al. [30]	2	2	1	1	**6**	Moderate
Soman et al. [35]	1	2	2	1	**6**	Moderate
Wu et al. [38]	2	2	1	1	**6**	Moderate
Zhang et al. [44]	1	1	2	2	**6**	Moderate
Zhou et al. [48]	2	1	2	1	**6**	Moderate
Zhou et al. [49]	2	0	1	3	**6**	Moderate
B. Luo et al. [11]	2	1	2	0	**5**	Moderate
M. R. Banik and T. Das [32]	2	1	2	0	**5**	Moderate
Soman et al. [36]	2	1	2	0	**5**	Moderate
Yang et al. [42]	3	1	1	0	**5**	Moderate
Zhou et al. [47]	2	1	2	0	**5**	Moderate
**Low Quality 0 to 4 points**						
Wang et al. [10]	0	1	2	1	**4**	Low
Zhou et al. [45]	1	1	2	0	**4**	Low

An analysis of the Quality Assessment scores reveals significant variability in the methodological rigor of the current literature, highlighting gaps in the transition from theoretical optimization to field deployment. Based on the cumulative scores (S_total), 18.4% (9 articles) achieved a “High Quality” rating. These studies represent the highest tier of evidence, exhibiting the strongest combined performance across validation rigor, structural complexity, reproducibility, and robustness. The majority of the literature, 77.6% (38 articles), fell into the “Moderate Quality” tier, while 4.1% (2 articles) were classified as “Low Quality.”

A detailed examination of the individual evaluation dimensions (Figure 7) provides deeper insights into the methodological characteristics of the reviewed literature. Performance was strongest in the Validation Method criterion, with 84% of studies achieving a score of 2 (53%) or 3 (31%), indicating a prevalent use of high-fidelity Finite Element Models or experimental validation. However, significant limitations persist in Reproducibility and Robustness. Specifically, 84% of the studies received a score of 2 for reproducibility, indicating that they generally provide clear mathematical formulations and parameter descriptions but do not provide open-source code, limiting independent replication.

Robustness represents the weakest analytical dimension in the reviewed studies. Among the reviewed studies, 18% received a score of 0, indicating no explicit consideration of environmental or measurement uncertainties, while 37% received a score of 1, reflecting only qualitative consideration of uncertainty without quantitative robustness assessment. Structural Complexity also reveals an important limitation: 47% of the studies were evaluated using small-scale structures, highly reduced models, or cases with limited demonstrated scalability (scores 0 or 1). This restricts the extent to which their reported performance can be generalized to large-scale, high-DOF bridge structures. Taken together, these findings highlight two distinct challenges in the current literature: limited validation under realistic uncertainty and insufficient demonstration of scalability to complex bridge models. Consequently, the subsequent technical synthesis places greater analytical emphasis on findings supported by studies with higher overall methodological quality.

### 3.2. Bibliometric Characteristics of the Reviewed Articles

In the collection of articles specifically addressing the optimal placement of sensors in bridges, the past 15 years were considered. As shown in Figure 8, although discrepancies may exist between the publication date and the actual execution of studies, a general trend of consistency in the annual number of publications can be observed, with notable peaks in 2022 and 2024. However, these absolute differences do not represent a significant increase in research activity, reflecting a limited number of studies in this field.

This consistency, illustrated in Figure 8, can be attributed to several factors. First, the inherent interdisciplinarity of the topic poses a challenge, as it requires advanced knowledge in both structural engineering and computer science. Additionally, recent advances in optimization may not have demonstrated benefits that justify the costs and efforts associated with their practical implementation. This consistency in the number of publications since 2010 reflects a mature research field where the focus has transitioned from proposing basic optimization rules to refining complex, multi-objective frameworks for large-scale infrastructures.

Figure 9 and Figure 10 illustrate the distribution of publications among the top 10 most prolific authors in the field of optimal sensor placement for SHM of bridges. The analysis highlights that Li, HN, Yi, TH, and Zhou, GD, are the most active contributors, each with a significant number of publications (7, 7, and 6 articles, respectively). This dominance suggests a strong research focus and sustained contributions from these authors in advancing methodologies and applications related to sensor placement.

These authors have frequently collaborated on topics such as optimization algorithms (e.g., genetic algorithms, firefly algorithms) and wireless sensor network configurations. Their collective work underscores the importance of interdisciplinary collaboration, blending civil engineering with computational optimization techniques. For instance, their joint contributions to articles such as “Optimal Wireless Sensor Network Configuration for Structural Monitoring Using Automatic-Learning Firefly Algorithm” [45] and “Wireless Sensor Placement for Structural Monitoring Using a Generalized Genetic Algorithm” [46] reveal a clear emphasis on leveraging advanced algorithms to address challenges in SHM.

Additionally, authors like B. Luo, L. Yi, and H. Wu, with three publications each, have concentrated on novel algorithms such as harmony search, cat swarm optimization, and reinforcement learning strategies, showcasing a commitment to exploring innovative techniques for improving sensor placement efficiency.

Interestingly, while some authors (e.g., Soman, RN, and Onoufriou, T) have relatively fewer publications, their work often addresses niche aspects like multi-type sensor placement or specific constraints in wireless sensor networks. This diversification of focus reflects the multifaceted challenges in SHM, from algorithmic development to practical deployment considerations.

In contrast to the analysis that includes both authors and co-authors, the graph focused exclusively on principal authors reveals a distinct perspective on individual contributions. For instance, while Zhou, GD, and Soman, RN, maintain a prominent position in both analyses, other authors like Li, HN, and Yi, TH, who lead the co-author analysis, do not exhibit the same prominence in the principal author graph. This suggests that, although Li, HN, and Yi, TH, are prolific collaborators, their role appears to be more focused on contributing as co-authors rather than leading projects. On the other hand, Zhou, GD, consistently stands out as a principal author, reflecting a sustained leading-author contribution to the field of optimal sensor placement. This contrast highlights the importance of examining contributions from different perspectives to identify the specific roles researchers play in advancing the field.

On the other hand, the distribution of publications, as shown in Figure 11, underscores China’s dominant role in advancing research on sensor placement optimization for SHM. The notable presence of the United States, although not matching the research efforts seen in China, underscores its active contribution to the field. Furthermore, the involvement of multiple countries, including European nations, demonstrates global interest in addressing the challenges of SHM through diverse approaches and methodologies.

To provide a comprehensive overview of the current landscape in sensor placement optimization (SPO), the data extracted from the systematic review was synthesized into the following figures. These visualizations categorize the 49 reviewed articles based on sensor types, algorithmic approaches, and research motivations.

As shown in Figure 12, there is a marked predominance of accelerometers, which are utilized in most of the studies. This confirms that vibration-based monitoring is the most frequently reported approach in the reviewed corpus. However, the significant presence of strain gauges and the emergence of fiber optic sensors reflect an increasing interest in capturing local structural responses to complement global modal data.

Regarding the computational methods, Figure 13 illustrates that biological algorithms (a branch of metaheuristics) and sequential methods are the most frequent choices. The overwhelming predominance of biological algorithms (present in 29 studies), such as Genetic Algorithms or the Firefly Algorithm, indicates a strong trend toward solving complex, high-dimensional, and multi-objective optimization problems that traditional numerical methods cannot easily address. Meanwhile, the prevalence of sequential methods (15 studies), such as Effective Independence, highlights the ongoing need for computationally efficient solutions. Furthermore, the emergence of AI-based methods (10 studies) reflects a growing shift toward intelligent and adaptive frameworks, which, alongside deterministic (9 studies) and physical algorithms (8 studies), complete the current methodological landscape.

The systematic extraction indicates a progressive shift in the underlying objectives driving OSP research. The descriptive definitions of each motivation category are established in the methodological framework (Table 6). However, the quantitative distribution of the literature (illustrated in Figure 14) reveals that maximizing data quality and monitoring accuracy is currently the most frequently identified motivation. While historical approaches prioritized minimizing initial capital expenditure (cost reduction via sensor count minimization), network lifespan and communication efficiency have emerged as additional drivers, although they remain minority motivations in the reviewed corpus. This transition highlights maturation in the field, indicating that modern SHM focuses on securing long-term, noise-free structural data over mere spatial distribution to effectively support autonomous Digital Twins.

**Table 6:** Categories and subcategories for analysis.

Category	Subcategory	Description
Type of algorithms	Biological algorithms	Algorithms that mimic natural processes such as evolution and animal behavior to solve complex problems, such as genetic algorithms or particle swarm optimization.
	Deterministic algorithms	Algorithms that follow a fixed set of rules and produce the same result whenever run under the same initial conditions, such as the EFI placement method.
	Sequential algorithms	Algorithms that process data or perform calculations in a specific, linear order, commonly used in optimization based on gradual or iterative steps.
	Physical algorithms	Algorithms inspired by physical phenomena, such as particle movement or simulated annealing, to optimize sensor placement in bridges.
	AI-based algorithms	Algorithms that employ artificial intelligence, such as machine learning or neural networks, to identify patterns and optimize solutions in complex systems.
Type of sensors	Accelerometer	Sensor used to measure the acceleration of an object. It is widely used in structural monitoring to detect vibrations and analyze the dynamic behavior of bridges.
	Strain Gauge	Sensors that measure the strain of a material. They are essential for assessing local changes in the structure and detecting potential damage or deterioration.
	Piezoelectric	Sensors that convert mechanical vibrations into electrical signals. They are useful for detecting structural damage and can also be used to harvest energy in environments with vibrations.
	Optical fiber	Sensors that measure strains and temperatures using light transmission through optical fibers, offering high precision and durability.
	GNSS	GNSS sensors provide precise 3D positioning, velocity, and time data, making them essential for monitoring structural displacements. They are highly accurate, capable of detecting static and dynamic shifts within millimeter precision.
	Seismometer	It is a sensor designed to measure ground motions, including vibrations caused by seismic activity, structural movements, or dynamic loads, providing critical data for assessing structural health and stability.
	Thermal sensors	Sensors that measure temperature variations in a structure, essential for understanding the impact of thermal changes on structural behavior.
Motivations	Cost reduction	Minimization of costs related to sensor installation and operation without compromising the quality of structural monitoring.
	Improvement in the quantity and placement of sensors	Optimization in the number of sensors and their placement to maximize data coverage and improve the effectiveness of structural monitoring.
	Communication efficiency	Optimization of wireless sensor networks to ensure efficient data transmission and minimize losses or interference in the monitoring system.
	Improvement in data quality and monitoring	Improvement in the accuracy and reliability of collected data through optimization techniques that reduce noise and errors in measurements.
	Optimization of network lifespan and energy efficiency	Extension of the lifetime of sensor networks through energy-efficient strategies and system designs that minimize resource consumption.

The analysis of research drivers, presented in Figure 14, reveals that improving data quality and monitoring effectiveness is the most frequent motivation in the field, driving 45 of the reviewed studies. While optimizing the quantity and placement of sensors remains a highly relevant objective (present in 33 studies), the current focus has increasingly shifted toward ensuring that the sensor network provides high information density for reliable structural health assessment. Furthermore, traditional operational constraints such as minimizing costs, enhancing communication efficiency, and optimizing energy consumption now hold a secondary but equal relevance, with each motivating 11 of the reviewed studies.

### 3.3. Results by Type of Sensor Category

A systematic synthesis of the extracted data demonstrates that sensor selection is closely linked to the target objective function and the scale of the infrastructure. Rather than operating in isolation, the highest-quality studies (representing 18.4% of the dataset) exhibit a clear transition toward heterogeneous networks. The analysis reveals that, while 46 of the 49 reviewed papers (94%) still rely on vibration-based global monitoring (accelerometers), the remaining subset driving the technological frontier incorporates localized instrumentation (strain gauges and optical fibers) to resolve the lack of spatial resolution inherent to macroscopic modal parameters. The specific implementation characteristics found in the literature for each modality are synthesized in the comparative evaluation presented in Section 4.3.

### 3.4. Results by Type of Algorithms Category

The extraction protocol reveals a clear change in the distribution of computational approaches used in OSP. The dataset demonstrates that deterministic approaches are primarily restricted to low-DOF theoretical models due to combinatorial explosion. In contrast, 80% of the studies applied to medium-to-large-scale bridge networks utilize metaheuristic or AI-based algorithms. This distribution suggests that metaheuristic and AI-based frameworks are increasingly used to address nonlinear and multi-objective constraints, such as noise and energy consumption in wireless nodes, which are difficult to handle simultaneously using classical sequential methods. The specific algorithmic paradigms identified in the literature are categorized using the proposed Sensor Placement Optimization Maturity Levels (SPO-ML):

- Level I (Basic-Static): Sequential methods (EFI) for structures with simple vibration modes or historic bridges where cost is the primary constraint.
- Level II (Advanced-Dynamic): Metaheuristic algorithms (GA, PSO) manage multiple objectives, ideal for long-span bridges and complex modal shapes.
- Level III (Intelligent-Adaptive): AI and DRL-based algorithms enabling self-configuring networks and real-time optimization against environmental changes.

The exhaustive conceptual descriptions of these algorithmic typologies are detailed in the methodological framework (Table 6), whereas their critical operational limits, computational costs, and specific real-world structural applications are systematically synthesized in the Unified Operational Decision Framework presented in Section 4.1.

### 3.5. Advances in Objective Functions for Optimal Sensor Placement

In the context of Structural Health Monitoring (SHM), the mathematical formulation of the objective function is a critical step, as it directly translates the physical, informational, and operational goals of the sensor network into an optimizable metric. Existing literature on OSP objective functions can be categorized into six major thematic groups: modal orthogonality, information and estimation theory, energy-based criteria, spatial redundancy, damage sensitivity and data reconstruction, and WSN-specific, multi-objective criteria.

#### 3.5.1. Modal Orthogonality and Independence Criteria

Ensuring that the extracted mode shapes are distinguishable from one another is a primary goal in OSP. The most widely adopted objective function in this category is the Modal Assurance Criterion (MAC). The objective is typically formulated as the minimization of the maximum off-diagonal elements of the MAC matrix, ensuring linear independence among the targeted mode shape vectors. To refine this approach, several authors express it as a fitness maximization problem as shown in Equation (1), ensuring the highest possible modal resolution during the optimization process [5,32]. This criterion has been extensively utilized in conjunction with metaheuristic algorithms such as the Chaotic Monkey Algorithm [44], Deep Reinforcement Learning [5], the COOT Algorithm [21], and various Firefly and Swarm Algorithms [11,18,23,27,29,47,48].

(1) $$f=1-max(MAC_{i,j})$$

Additionally, the Singular Value Decomposition Ratio (SVDR) is utilized to measure mode orthogonality. By minimizing the ratio of the maximum to the minimum singular values of the modal matrix, the SVDR improves the conditions for mode expansion and system observability [45,46].

#### 3.5.2. Information and Estimation Theory Criteria

Derived from parameter estimation theory, the Fisher Information Matrix (FIM) quantifies the amount of information that observable variables carry about the unknown structural parameters. The Effective Independence (EFI) method aims to maximize the determinant, trace, or fractional rank of the FIM to minimize the covariance of the estimation error, thereby reducing the sensitivity of the estimates to ambient noise [4,25,26,33]. This criterion has been successfully applied to tailor experimental setups for historical bridges [33] and complex civil structures using relaxed convex optimization [22,37]. Advanced variations have also been developed, such as the Triaxial Effective Independence (TEI) to handle 3-axis sensors efficiently [19,34], and the Sensitivity-EFI method, which incorporates damage sensitivity coefficients directly into the FIM formulation to account for structural mass and stiffness redistributions [12,28].

Rooted in Bayesian statistics, Information Entropy (IE) measures the uncertainty in the estimation of structural parameters. The objective is to minimize the asymptotic information entropy and maximize the posterior probability of detecting structural changes. This metric is highly effective for handling operational uncertainties, such as unknown ship-collision locations [24] and environmental measurement noise [49].

#### 3.5.3. Energy-Based and Signal-to-Noise Criteria

Energy-based methods rely on the physical premise that sensors should be placed at degrees of freedom (DOFs) exhibiting the highest dynamic energy. The Modal Strain Energy (MSE) and Modal Kinetic Energy (MKE) are frequently maximized to ensure that the sensors capture prominent structural responses, naturally improving the signal quality [2,14,25,38,50]. Recently, MSE has been reformulated as a Quadratic Unconstrained Binary Optimization (QUBO) problem to be solved using Quantum Computing algorithms [1].

To explicitly address environmental and channel noise, the Modal Signal-to-Noise Ratio (MSNR) has been proposed. By maximizing the minimum MSNR across targeted modes, the sensor placement ensures that the acquired signals strongly outweigh the background noise, facilitating the reliable extraction of modal parameters even under low-excitation conditions [15].

#### 3.5.4. Spatial Correlation and Redundancy Criteria

To avoid sensor clustering and ensure a broad spatial distribution of the monitoring network, Information Theory metrics such as Mutual Information (MI) and the Correlation Matrix of Responses (CMR) are introduced as objective functions. Minimizing MI or the off-diagonal elements of the CMR aims to ensure that each deployed sensor provides independent, non-redundant information [16,43]. Similarly, the Information Redundancy Rate (FRE) and Information Entropy Index (IEI) are used as penalty functions to evaluate the overlap of information between candidate locations, avoiding unnecessary instrumentation costs [19,34].

#### 3.5.5. Damage Sensitivity and Data Reconstruction Criteria

Shifting the focus from global modal properties to localized degradation, Damage Sensitivity Indexes (DSI) have been proposed to maximize the probability of detecting stiffness reductions in critical elements [12,43]. In this line, specific coverage metrics such as the Strain Difference Coverage (SDC) and Strain Contribution Coverage (SCC) have been developed to evaluate if a placed sensor can detect strain value changes under various load cases and damage scenarios [38,50], and sensitivity-based sensor placement strategies have been specifically validated on steel truss bridges [54].

Furthermore, when the objective of the sensor network is Virtual Sensing (estimating responses at unmeasured locations), the Minimum Mean Square Error Criterion (MSEC) or Root Mean Square Error (RMSE) is adopted. This function minimizes the variance of the estimation error between the actual structural response and the response reconstructed using techniques like Kalman Filtering or Artificial Neural Networks, ensuring high-fidelity full-field dynamic behavior reconstruction [12,13,16,17,31].

#### 3.5.6. Multi-Objective and WSN-Specific Criteria

With the large-scale deployment of Wireless Sensor Networks (WSNs), the OSP problem has evolved to incorporate multidisciplinary objectives. In these formulations, civil engineering metrics are weighted against computer science constraints. A prominent functional objective aims to maximize the ratio of the FIM determinant to the maximum energy consumed by a node (f=∥Q∥/E), ensuring high-quality data per unit of energy [6,35,36]. Additionally, complex multi-objective frameworks have been designed to simultaneously maximize modal linear independence while ensuring network connectivity, optimize data routing paths, and maintain energy balance across the sensor nodes to prolong the system’s lifespan [40,41,45,47,48]. For post-event rapid assessments, logistical constraints have also been integrated as objective functions, such as minimizing the number of non-detectable damage scenarios, maximizing damage detectability, and minimizing the average time-to-detection [7].

### 3.6. Co-Occurrence Patterns Across Algorithms, Sensors and Motivations

To move beyond a descriptive repository of the literature, a co-occurrence analysis was performed on the extracted data matrix (Figure 15). By examining how frequently algorithm typologies, sensor modalities, and primary motivations appear together within the same study, three recurring co-occurrence patterns can be identified. These patterns are descriptive rather than statistically inferred and are presented as observed tendencies in the reviewed corpus rather than as formally derived clusters.

Cluster 1: The Traditional Dynamic Paradigm. The data show a frequent co-occurrence of deterministic and sequential algorithms (e.g., EFI) with the predominant use of accelerometers and a primary motivation for cost reduction. This cluster represents the classical approach to SHM, where the objective is to find a mathematically exact minimum number of sensors for global modal identification. However, the quality assessment indicates that this cluster is increasingly confined to small-scale or historical structures due to scalability limitations.

Cluster 2: The High-Dimensional Multi-Objective Paradigm. A second, dominant cluster demonstrates a high co-occurrence between metaheuristic algorithms (biological and physical), heterogeneous sensor networks (accelerometers combined with strain gauges or optical fibers), and the dual motivation of improving data quality and communication efficiency. This pattern suggests that as structures grow in complexity (e.g., long-span cable-stayed bridges), researchers tend to replace exact deterministic methods with swarm intelligence to solve multi-objective, high-dimensional combinatorial problems.

Cluster 3: The Smart Autonomous Paradigm. Finally, the data highlights an emerging third cluster mainly associating AI-based algorithms (such as Deep Reinforcement Learning) with Wireless Sensor Networks (WSNs), focusing heavily on optimizing network lifespan and energy efficiency. This cluster represents the frontier of SHM, marking a shift from static sensor placement to dynamic, energy-aware IoT scheduling, where algorithmic adaptability is prioritized over traditional mathematical optimality.

### 3.7. Synthesis of Findings Against Research Questions

To provide cohesive closure to the systematic extraction, the quantitative and categorical data are synthesized below to directly address the core objectives driving this review.

Regarding the optimal strategies for sensor placement, the extracted data indicates that the suitability of each algorithm is closely associated with the structural scale. For low-DOF or historical structures, deterministic sequential methods (e.g., Effective Independence) remain the most utilized due to their computational efficiency. Conversely, for large-scale infrastructures, the reviewed literature increasingly favors hybrid metaheuristics (e.g., GA, Firefly) and Artificial Intelligence models (e.g., DRL). These frameworks are particularly well suited to addressing high-dimensional, multi-objective optimization problems in which conventional sequential approaches become computationally restrictive.

In terms of evaluation, most strategies were assessed through finite element simulations (26 studies), whereas 15 studies included experimental or field validation of physical bridges (Section 3.1).

In terms of dominant sensor modalities, accelerometers represent the dominant sensor modality, utilized in 46 of the 49 studies (94%) due to their robustness in extracting global modal parameters. However, the data reveals that the highest-quality monitoring frameworks deploy heterogeneous networks. Optimal configurations position accelerometers at global modal anti-nodes, while strategically placing localized sensors at high-stress joints to compensate for the accelerometer’s low sensitivity to early-stage material yielding.

Finally, analyzing the fundamental motivations for optimization, the literature indicates a clear evolution. While economic cost reduction through minimal sensor counts was the historical driver, contemporary research is predominantly motivated by maximizing data quality and monitoring effectiveness, identified in 45 of the 49 reviewed studies. Network lifespan, communication efficiency and cost reduction each motivate 11 studies, indicating that energy-aware and transmission-oriented objectives, although central to the emerging Wireless Sensor Network paradigm, are not yet dominant across the corpus.

Regarding the second part of RQ3, the benefits of OSP are predominantly demonstrated numerically. According to the Validation Method dimension (Table 5, Figure 7), only 15 of the 49 studies (31%) verified their configurations experimentally on a physical bridge or through a full-scale field deployment. Moreover, reported improvements are expressed through study-specific indices, such as off-diagonal MAC values, the FIM determinant, information entropy or response reconstruction error, computed for different structures, candidate sets and sensor budgets; consequently, they cannot be aggregated into a common magnitude of benefit. Only 22 studies quantitatively evaluated performance under at least one source of uncertainty (Robustness score ≥ 2). Therefore, the most frequently coded motivations should be interpreted as research objectives, while field-demonstrated benefits remain limited within the reviewed corpus.

Building upon these empirical results, the qualitative relationships observed across the literature provide the foundation for an operational guideline, particularly the correlation between structural complexity (DOFs), computational cost limits, and sensor network constraints. Rather than presenting isolated algorithmic preferences, the extracted data suggests a hierarchical selection pattern. These synthesized observations are consolidated into the Unified Operational Decision Framework, which is presented and discussed in Section 4.1, thereby fulfilling the primary applied objective of this review.

## 4. Discussion

The results obtained from this systematic review highlight significant trends and evolutionary shifts in the field of sensor placement optimization (SPO) for bridge structures. This section interprets these findings, addressing technical implications and the current gaps identified in the literature.

An interpretive framework is proposed, linking civil engineering objectives with computer science capabilities. This model establishes that algorithm selection must be a balanced function between Information Fidelity (Civil) and Computational Cost (CS). While civil engineers prioritize modal parameter accuracy, the viability of real SHM depends on energy and network efficiency, especially in WSNs.

### 4.1. A Unified Operational Framework for Engineering-Oriented Algorithm Selection

The interdisciplinary nature of SHM demands a mathematically rigorous yet practically viable approach to algorithm selection. The synthesized literature demonstrates that no single optimization paradigm is universally superior; rather, algorithmic efficacy is strongly associated with structural complexity, typically measured in Degrees of Freedom (DOFs), and specific operational constraints. To bridge the gap between computational theory and civil engineering deployment, we propose a unified decision framework that translates these observed patterns into practical operational guidance for OSP implementation.

Algorithmic selection can be guided by the following synthesized operational patterns, derived from the distribution of algorithmic choices observed across the reviewed literature (Section 3.4) and expressed using the same complexity bands used in the Quality Assessment classification (Table 4):

- Small-Scale and Heritage Range (DOFs < 1,000, indicative): For dimensionally reduced models or historical structures where budget preservation is critical, the reviewed literature shows a consistent preference for deterministic and sequential methods (e.g., Effective Independence), which offer transparent formulations and require minimal computational overhead.
- Large-Scale Complexity Range (DOFs >= 10,000, indicative): As structures approach this scale (e.g., long-span cable-stayed bridges), the reviewed studies increasingly report sequential methods becoming computationally impractical due to combinatorial explosion. In this segment, hybrid metaheuristics (e.g., Genetic Algorithms, Particle Swarm Optimization) dominate the literature, navigating massive search spaces while avoiding premature convergence to local optima.
- Energy and Lifespan Constraint: In scenarios dictating that a Wireless Sensor Network (WSN) must operate autonomously for extended lifecycles, purely structural objective functions (such as the Modal Assurance Criterion) are insufficient. The operational threshold necessitates multi-objective algorithms (e.g., Firefly Algorithm or AI-based models) that maximize the Information-to-Energy ratio (f = ‖Q‖/E), balancing optimal data routing paths with minimal node energy consumption.

These synthesized operational patterns are consolidated into the Unified Decision Matrix (Table 7) and visualized as a decision-tree framework in Figure 16, providing a practical, literature-informed reference for algorithm selection. As defined in Section 2.3, the DOF ranges used in this framework are heuristic order-of-magnitude categories introduced for comparative synthesis rather than independently validated engineering limits. Their use here ensures consistency with the structural complexity classification applied in the Quality Assessment. The exact crossover between algorithmic paradigms remains problem-dependent and may vary with factors such as candidate sensor density and computational resources.

From an engineering perspective, these results suggest that the selection of an OSP strategy should be driven primarily by the monitoring objective, structural complexity, and operational constraints rather than by the theoretical performance of the optimization algorithm alone. For conventional modal monitoring of small or moderately complex structures, sequential methods such as Effective Independence remain an appropriate first choice because of their low computational requirements, transparent formulation, and ease of implementation. When the number of candidate locations and structural degrees of freedom increases, particularly in long-span bridges, metaheuristic approaches become more suitable because they can explore large combinatorial search spaces and accommodate multiple objectives. AI-based approaches are most appropriate when the monitoring system requires adaptive operation, dynamic sensor scheduling, or integration with continuously updated Digital Twin environments. However, their higher training requirements, reduced interpretability, and dependence on representative training data must be considered before field deployment. Therefore, increasingly sophisticated algorithms should not be interpreted as universally superior alternatives but as tools whose practical suitability depends on the complexity and operational requirements of the monitoring problem.

### 4.2. Methodological Tensions and Algorithmic Limitations

While the synthesized literature delineates a trajectory of continuous algorithmic refinement in OSP, a rigorous audit of the extracted data reveals fundamental methodological discordances and unresolved boundary limitations.

The Deterministic vs. Metaheuristic Dichotomy: A primary analytical tension persists in the selection of optimization architectures for large-scale Structural Health Monitoring (SHM). Sequential approaches such as the Effective Independence (EFI) method provide transparent, low-cost formulations with fully repeatable outputs, but their greedy elimination scheme discards candidate degrees of freedom irreversibly and therefore converges to local rather than global optima. Exhaustive deterministic search does guarantee global optimality yet becomes computationally intractable at the high Degree of Freedom (DOF) density characteristic of contemporary Finite Element Models (FEM), where the combinatorial size of the candidate set grows prohibitively. Conversely, while literature frequently defaults to hybrid metaheuristics (e.g., GA, PSO) to circumvent these dimensionality constraints, a methodological vulnerability remains systematically underreported: stochastic instability. Metaheuristic frameworks provide no optimality guarantees, exhibit a propensity for premature convergence, and yield different configurations across independent runs, yet the reviewed studies rarely report run-to-run variability or convergence statistics. Furthermore, the reported computational efficiency of these stochastic methods is frequently offset by the unquantified overhead required for empirical hyperparameter calibration, a burden that is seldom included in the reported execution times. Emerging alternatives, such as partial-model-based strategies employing stiffness separation to avoid full-structure reconstruction, have shown promise in mitigating this computational burden for long-span steel truss bridges [55].

Vulnerabilities in Contemporary AI Deployments: A secondary methodological tension emerges from the proliferation of Deep Reinforcement Learning (DRL) and broader Artificial Intelligence paradigms within OSP. Despite being positioned as state-of-the-art, a recurrent limitation in the validation of these models is their frequent reliance on idealized, noise-free FEM data. Consequently, these frameworks are acutely susceptible to the simulation-to-reality gap. The extracted data indicates a recurrent tension: models demonstrating high theoretical accuracy in topological optimization may exhibit substantial performance degradation when subjected to real-world epistemic uncertainties (e.g., unmodeled structural dynamics) and aleatoric environmental noise. The prevailing discourse often treats AI architectures as agnostic panaceas, minimizing the implications of their inherent non-interpretability (“black-box” nature) and extreme fragility outside their specific training manifold.

Discordance in Objective Function Formulation: Finally, the systematic extraction indicates a persistent conflict regarding the mathematical formulation of objective functions. A substantial subset of the historical literature operates on the premise that minimizing sensor density linearly correlates with optimal cost-efficiency. However, contemporary empirical deployments contradict this paradigm. Sparse network topologies, while reducing initial capital expenditure, fundamentally compromise the spatial resolution of the monitoring array, which may increase false-negative rates for early-stage damage detection. The prevailing tension lies in the inability of current multi-objective frameworks to mathematically reconcile the deterministic cost of sensor deployment against the probabilistic, high-magnitude risk associated with undetected structural yielding.

### 4.3. Qualitative Comparison of Sensor Modalities

While the descriptive results highlight the individual utilization of each sensor type, a critical evaluation of the literature reveals distinct trade-offs regarding cost, data fidelity, and structural applicability, as summarized in Table 8. Accelerometers remain the most widely used modality in the reviewed studies, owing to their high frequency-response range and ease of global modal extraction; however, studies report that their relative performance degrades in detecting localized, low-energy damage, where signal-to-noise ratios drop significantly. Conversely, strain gauges and optical fibers offer superior localized sensitivity to material yielding but require dense deployment, substantially increasing installation complexity and network vulnerability.

Consequently, the reviewed evidence increasingly supports heterogeneous sensor deployments when both global and localized structural responses must be captured. Hybrid multi-type sensor networks can combine accelerometers for global modal information with localized sensing technologies at critical structural regions. A commonly supported configuration consists of deploying accelerometers at global modal anti-nodes to capture overarching mode shapes, supplemented by optical fibers or piezoelectric sensors at high-stress concentrations (e.g., cable anchorages or mid-spans).

From a practical instrumentation perspective, accelerometers therefore represent the most appropriate baseline solution when the primary objective is global modal identification or the detection of global stiffness changes, particularly in long-span structures. However, they should not be considered sufficient when the monitoring objective involves localized damage or early-stage material degradation. In such cases, strain gauges or optical fiber sensors provide complementary local information, whereas piezoelectric sensors are more suitable for high-frequency phenomena and crack-related monitoring. GNSS becomes relevant when the target variable is absolute displacement rather than local strain or modal response. Consequently, the reviewed evidence supports selecting sensor technology according to the physical quantity and spatial scale of the expected structural response, with heterogeneous sensor networks becoming preferable when both global and local damage indicators are required.

### 4.4. Historical Evolution of OSP Strategies and Research Motivations

The reviewed literature suggests three broad stages in the evolution of OSP for bridge SHM. Early studies during the 2010–2013 period were predominantly centered on modal-information criteria, sequential or deterministic optimization, and the reduction in sensor quantity while preserving structural observability [4,6,20,22,25,37]. From the mid-2010s onward, the increasing adoption of metaheuristic algorithms and wireless sensor networks expanded the OSP problem beyond sensor location alone, incorporating network connectivity, energy consumption, and multi-objective optimization [2,18,35,36,45,46,47,48]. More recent studies have progressively introduced heterogeneous sensing, data reconstruction, artificial intelligence, reinforcement learning, and Digital Twin-oriented monitoring [5,17,31,40,43]. This progression indicates that OSP has evolved from predominantly static sensor-number and modal-information optimization towards increasingly integrated structural and operational decision problems.

Historically, the primary motivation for optimization was the minimization of economic costs by reducing the total number of sensors. However, current findings suggest that contemporary research is increasingly driven by data quality and reconstruction accuracy. The focus has shifted toward ensuring that the sensor network provides sufficient information for high-fidelity Structural Health Monitoring (SHM). This indicates a maturation of the field; the objective is no longer simply to install “fewer sensors,” but rather to achieve the “optimal information density” required to support reliable decision-making in bridge maintenance and safety.

### 4.5. Overcoming the Simulation-to-Reality Gap: Limitations and Future Trajectories

Despite the profound algorithmic sophistication documented in the recent literature, a recurrent methodological limitation persists: the over-reliance on idealized, noise-free Finite Element Models (FEM) for algorithm validation. The predominant limitation in the current state-of-the-art is the simulation-to-reality gap. Algorithms optimized under deterministic, laboratory-controlled conditions may exhibit substantial performance degradation when subjected to aleatoric environmental noise, thermal fluctuations, and unmodeled structural non-linearities.

Consequently, the future trajectory of OSP research must pivot decisively from static topological optimization toward Robustness under Uncertainty. Next-generation frameworks must integrate stochastic noise injection and potential sensor failure directly into the mathematical formulation of their objective functions. Furthermore, the transition toward continuous digital twin operations necessitates replacing static sensor deployments with dynamically adaptive networks. In this context, Physics-Informed Machine Learning and Deep Reinforcement Learning represent promising computational approaches for adaptive OSP. These AI-driven paradigms can potentially support continuous prediction and adjustment of sensor configurations in real time, effectively responding to the progressive degradation of the civil infrastructure over its operational lifecycle.

In addition, the comparability of OSP studies is limited by heterogeneous reporting of the monitored structures. Future studies will provide greater engineering value by consistently reporting, at a minimum, the bridge typology and main span, the size of the finite element model and of the candidate sensor set, the number, type and location of the selected sensors, the target modes and their natural frequencies, the damping assumptions, and whether performance was verified with field measurements. Such standardized reporting would enable future reviews to compare sensor configurations and dynamic characteristics across bridges, which was beyond the scope of the present bibliometric and methodological synthesis.

### 4.6. Practical Recommendations for Structural Engineers

Based on the synthesized evidence, OSP should not begin with the selection of an optimization algorithm, but with the definition of the monitoring objective and operational constraints. For global modal identification in reduced or moderately complex structural models, sequential approaches such as EFI remain suitable due to their low computational demand and straightforward implementation. For long-span bridges with large candidate sensor sets or multiple competing objectives, metaheuristic approaches provide greater scalability. AI-based methods become particularly relevant when adaptive operation, WSN scheduling, or continuous Digital Twin integration is required, although their use should be supported by explicit robustness validation. Regarding instrumentation, accelerometers remain appropriate for global modal monitoring, whereas strain-based, optical, or piezoelectric sensing should complement them when localized damage detection is required. Therefore, sensor modality, algorithmic paradigm, and validation strategy should be selected jointly rather than independently.

## 5. Conclusions

This systematic review critically evaluated 49 included studies to synthesize the state-of-the-art in Optimal Sensor Placement (OSP) for Structural Health Monitoring (SHM) in bridges. By systematically correlating algorithmic typologies, sensor modalities, and operational motivations, this research bridges the persistent interdisciplinary gap between structural engineering constraints and computer science optimization capabilities.

The findings indicate a progressive shift in structural monitoring. The historical reliance on homogeneous sensor arrays and purely deterministic mathematical methods, while foundational, is increasingly inadequate for the dimensional complexity of modern infrastructure. To address this, this study proposes the Sensor Placement Optimization Maturity Levels (SPO-ML) and a Unified Operational Decision Framework. These practical tools establish that algorithmic selection cannot be arbitrary; it should be scaled to structural complexity (Degrees of Freedom) and constrained by real-world parameters, such as the energy limitations of Wireless Sensor Networks (WSNs).

Furthermore, the analysis reveals that contemporary OSP is no longer driven merely by the economic desire to minimize sensor counts. Recent studies increasingly prioritize data fidelity to support the deployment of autonomous Digital Twins. Consequently, the reviewed evidence increasingly supports heterogeneous networks, strategically combining global accelerometers with localized strain gauges or optical fibers to capture comprehensive structural behavior.

Ultimately, this review exposes a critical methodological vulnerability in the current literature: the simulation-to-reality gap. The substantial performance degradation of AI-based and metaheuristic algorithms when they are transitioned from idealized numerical models to noisy, real-world environments remains a primary challenge. Future research trajectories must evolve beyond static topological optimization, prioritizing stochastic robustness, real-time algorithmic adaptability, and energy-aware IoT scheduling. Only by mathematically integrating these real-world uncertainties can optimization algorithms transition from academic simulations into reliable, field-deployable solutions for global civil infrastructure.

## Figures and Tables

**Figure 1:**
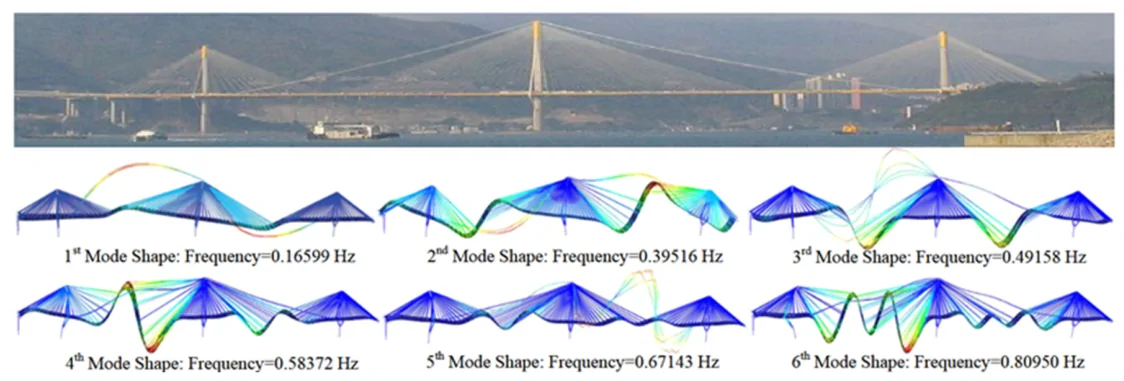
Global vibration mode shapes of the Ting Kau Bridge as structural information for modal-based sensor placement strategies [6].

**Figure 2:**
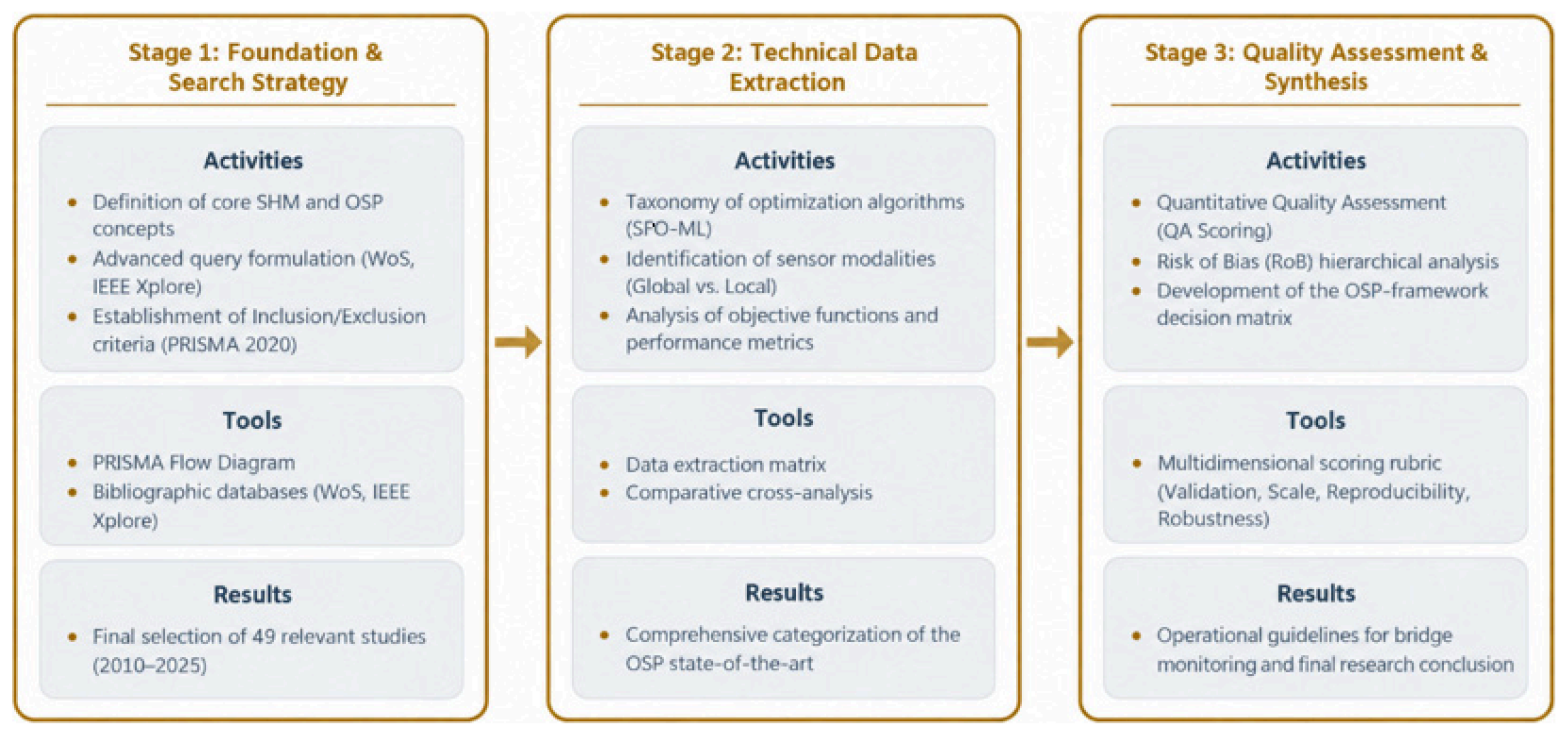
Systematic Research Methodology Framework.

**Figure 3:**
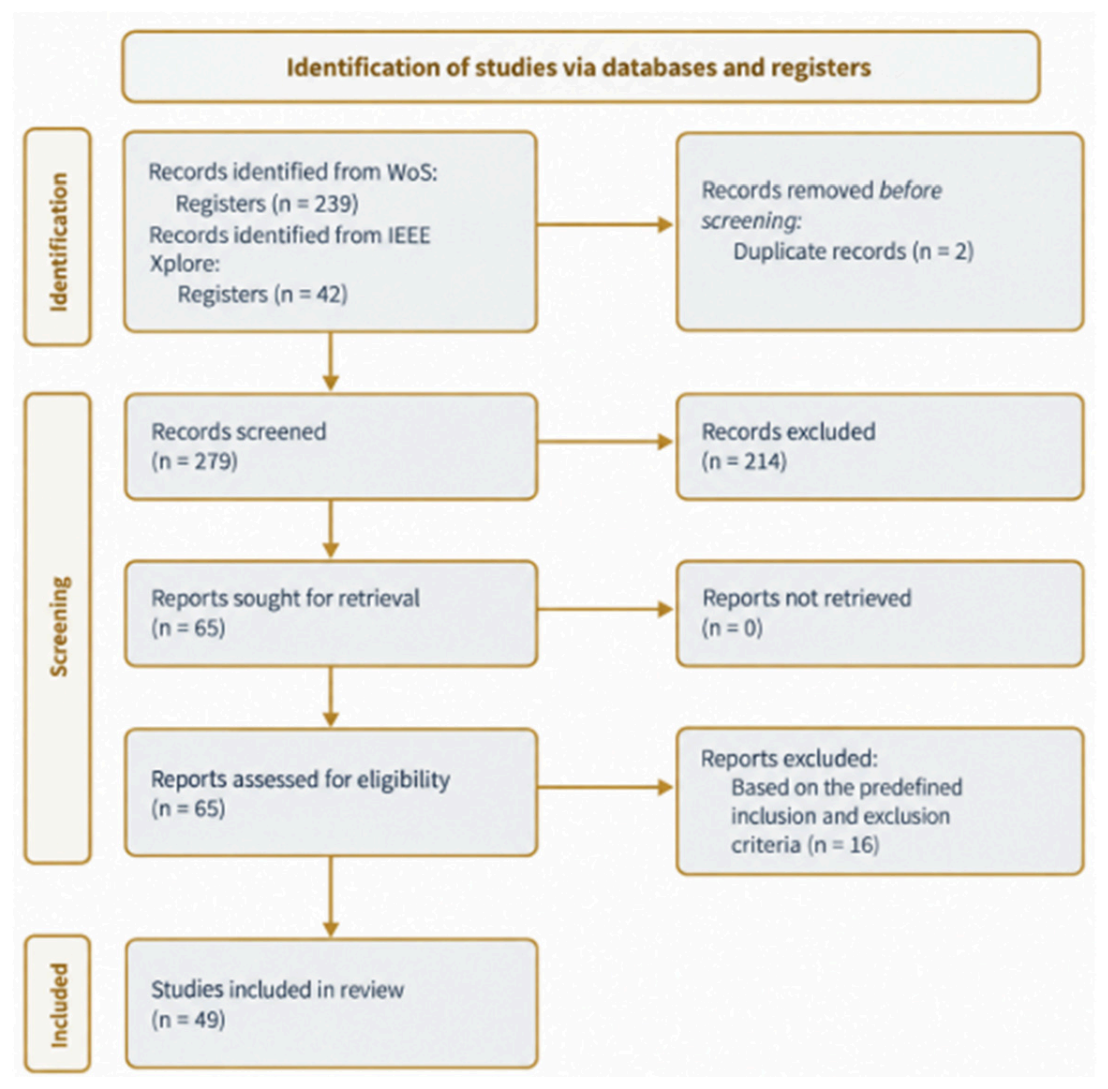
Flowchart of the Article Selection Process.

**Figure 4:**
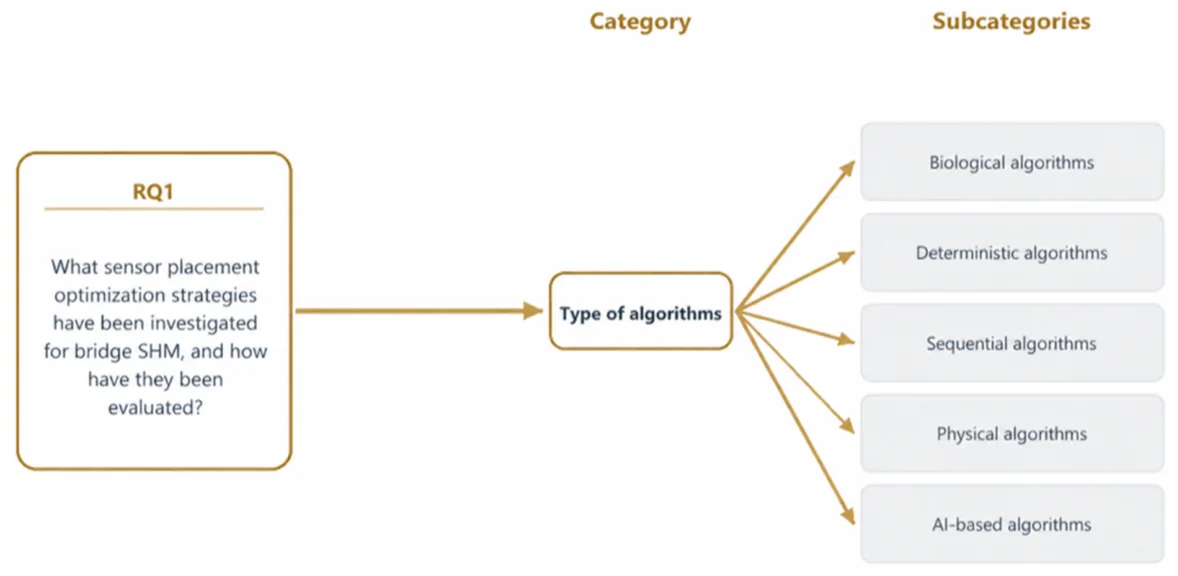
Criteria and subcategories for research question 1.

**Figure 5:**
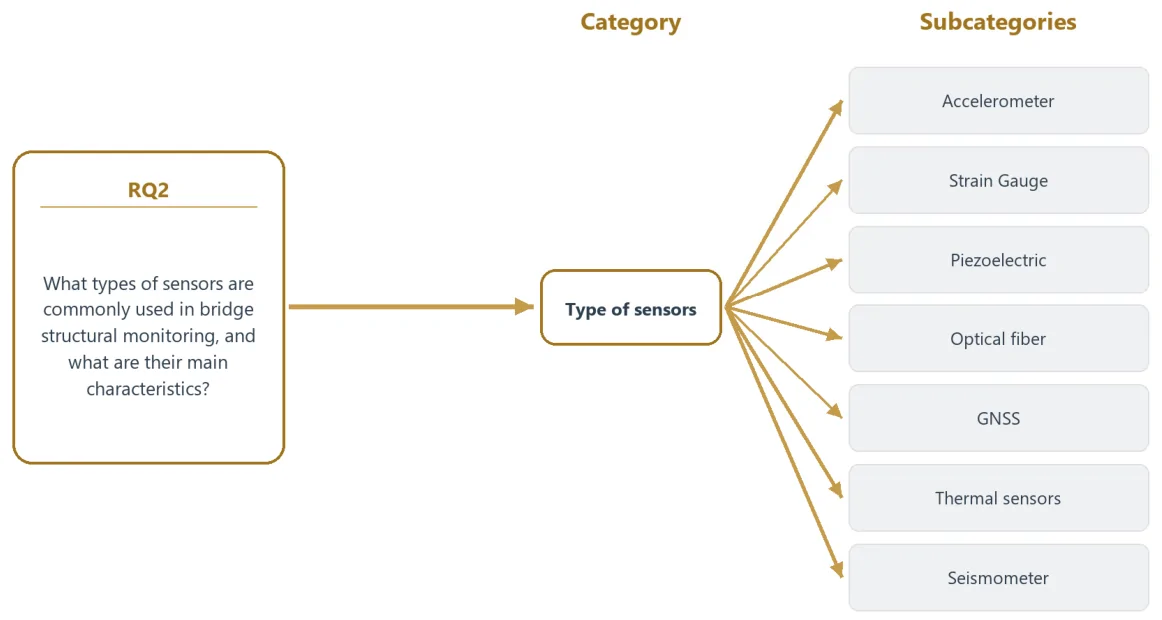
Criteria and subcategories for research question 2.

**Figure 6:**
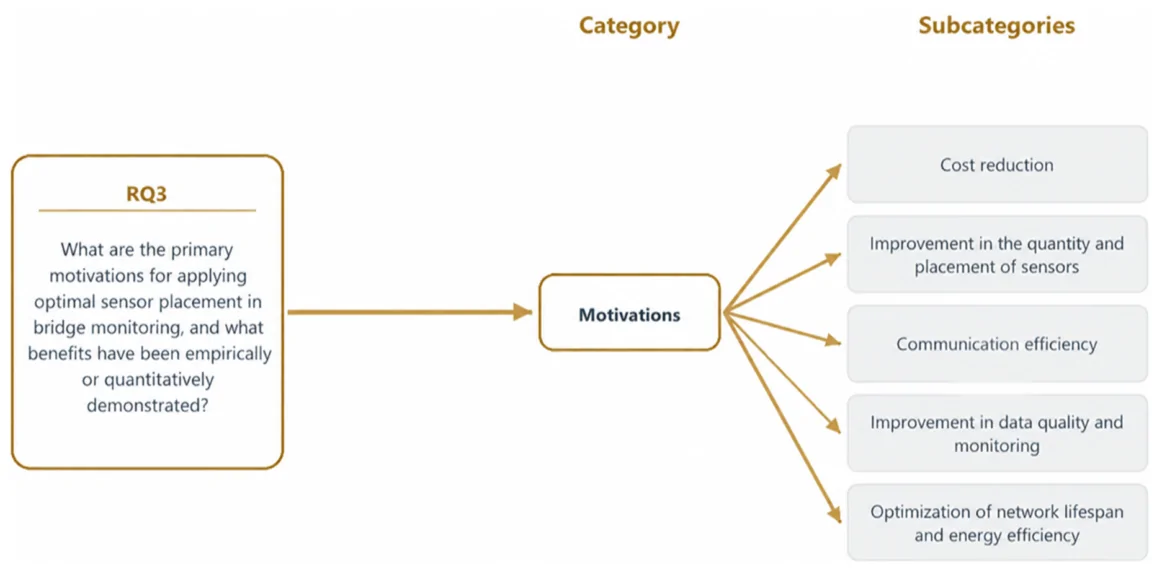
Criteria and subcategories for research question 3.

**Figure 7:**
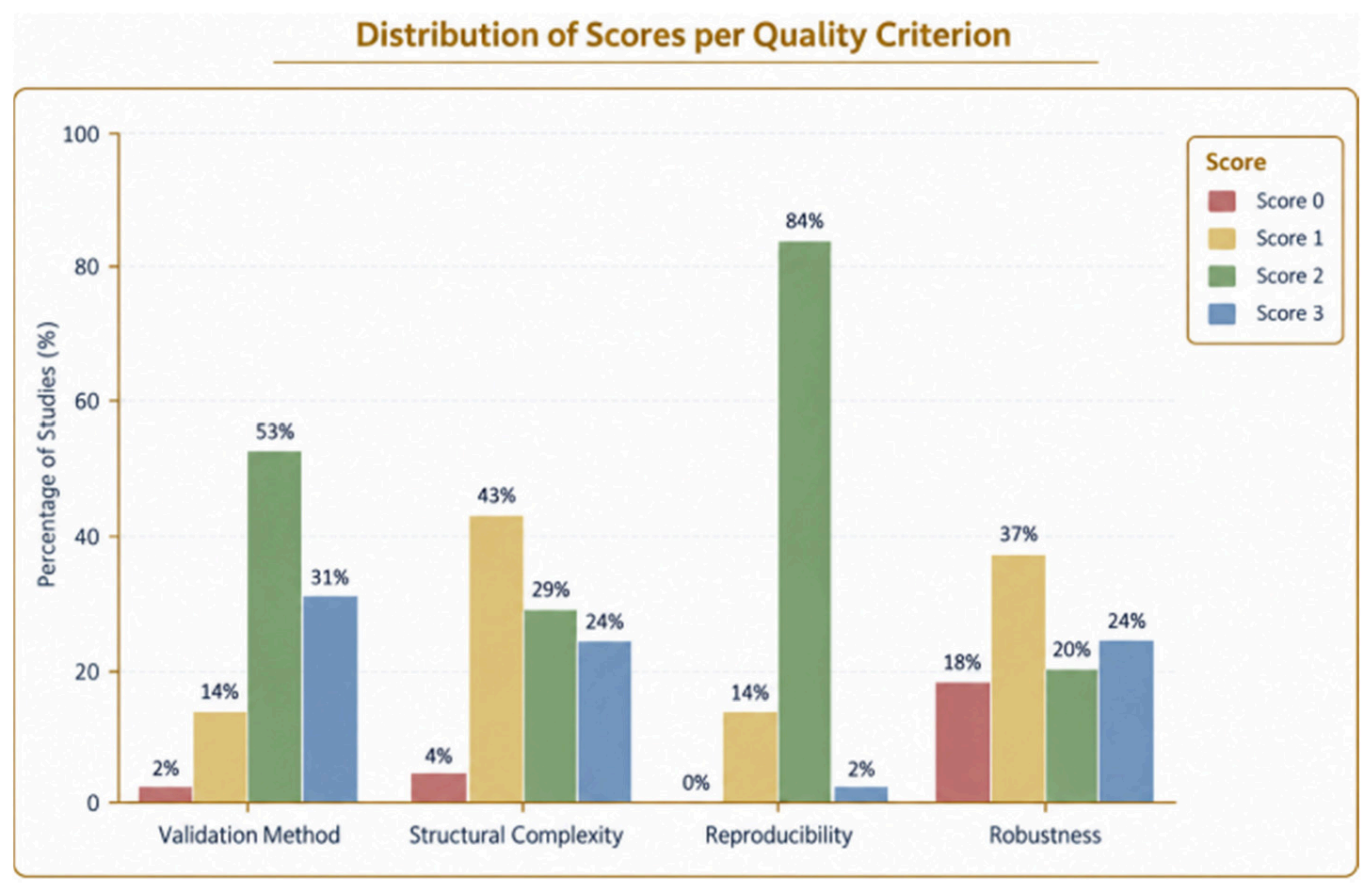
Distribution of quality assessment scores across the four evaluated technical dimensions.

**Figure 8:**
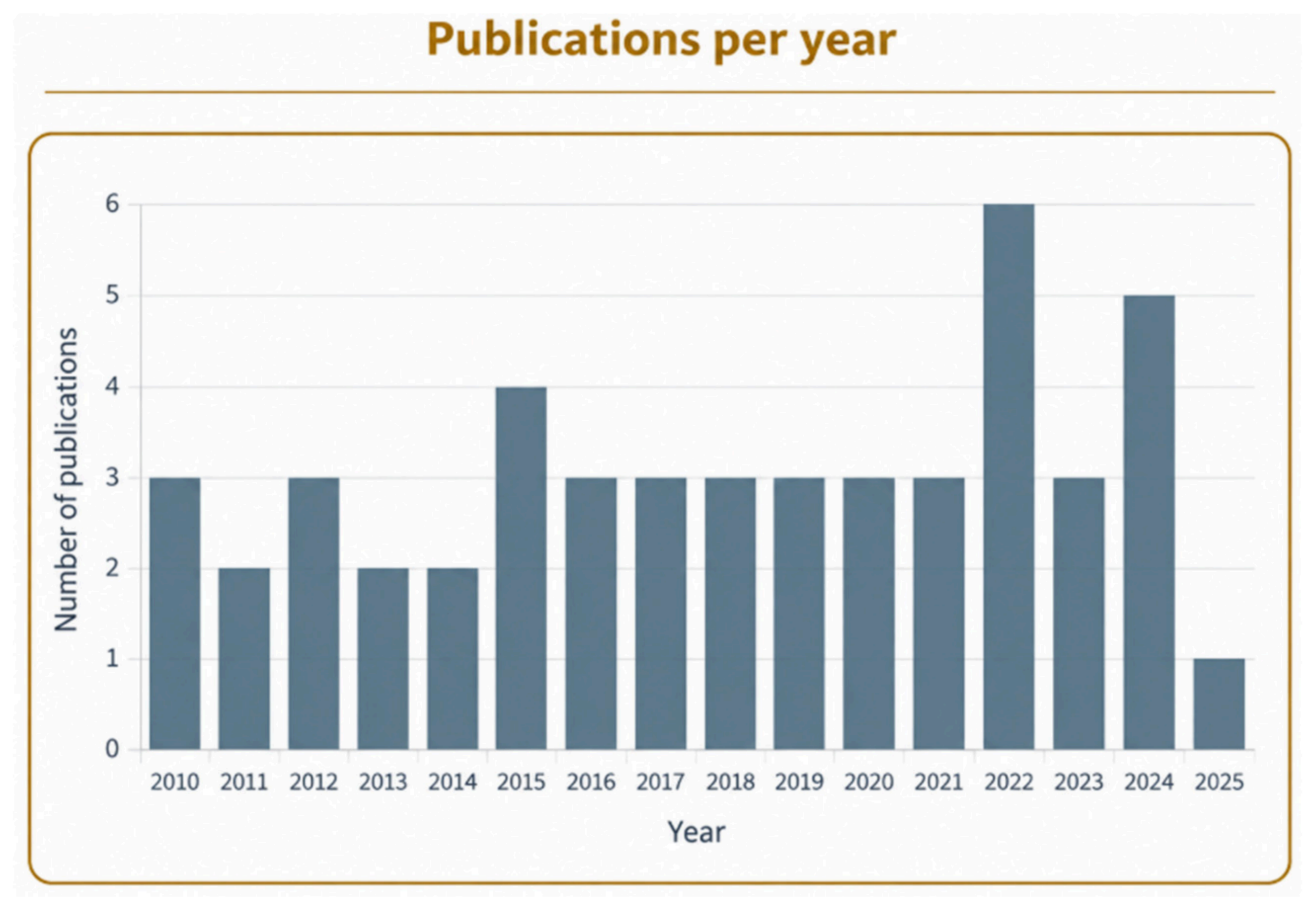
Number of articles per year.

**Figure 9:**
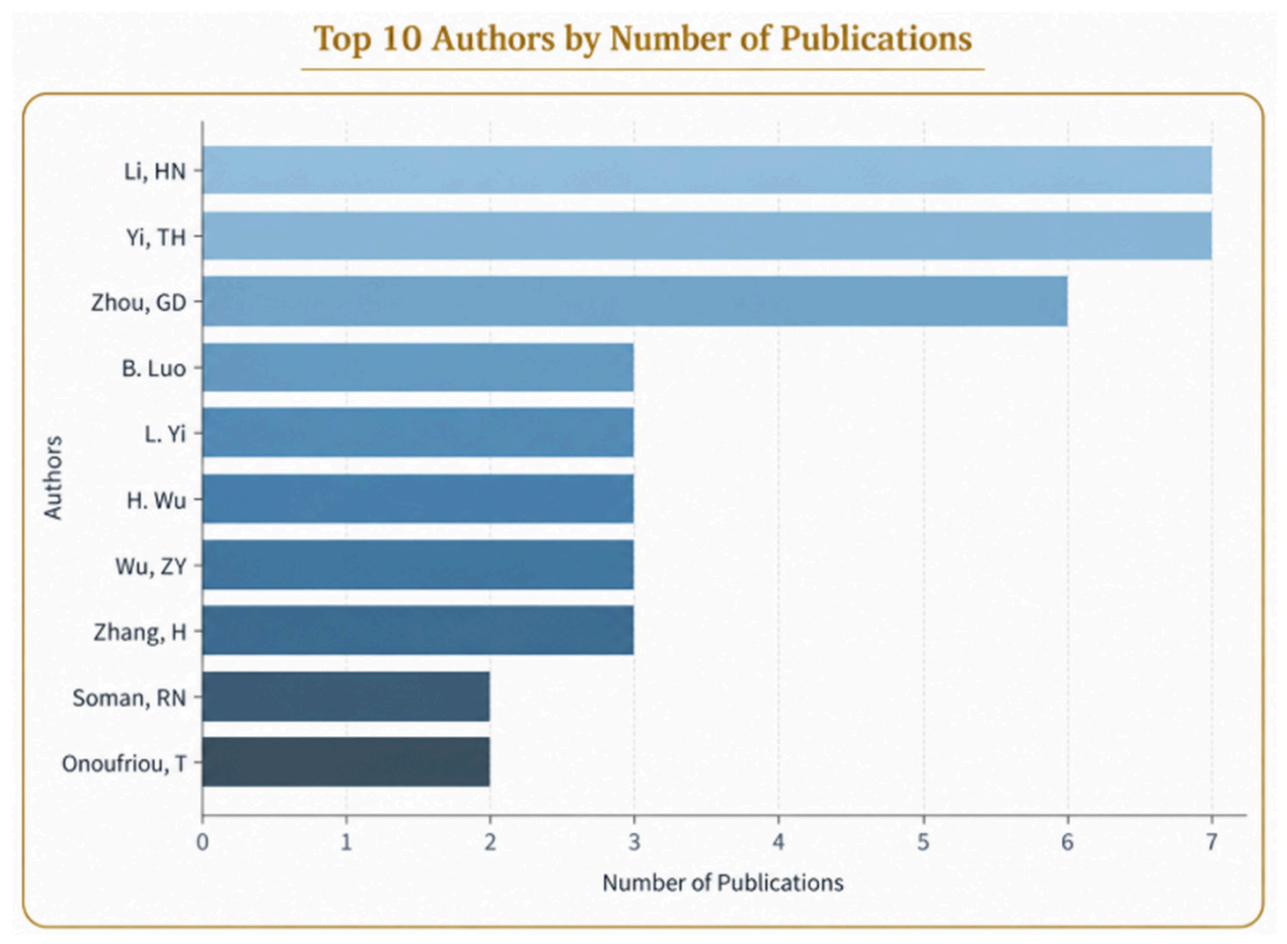
Top 10 authors by number of publications (including co-authorship).

**Figure 10:**
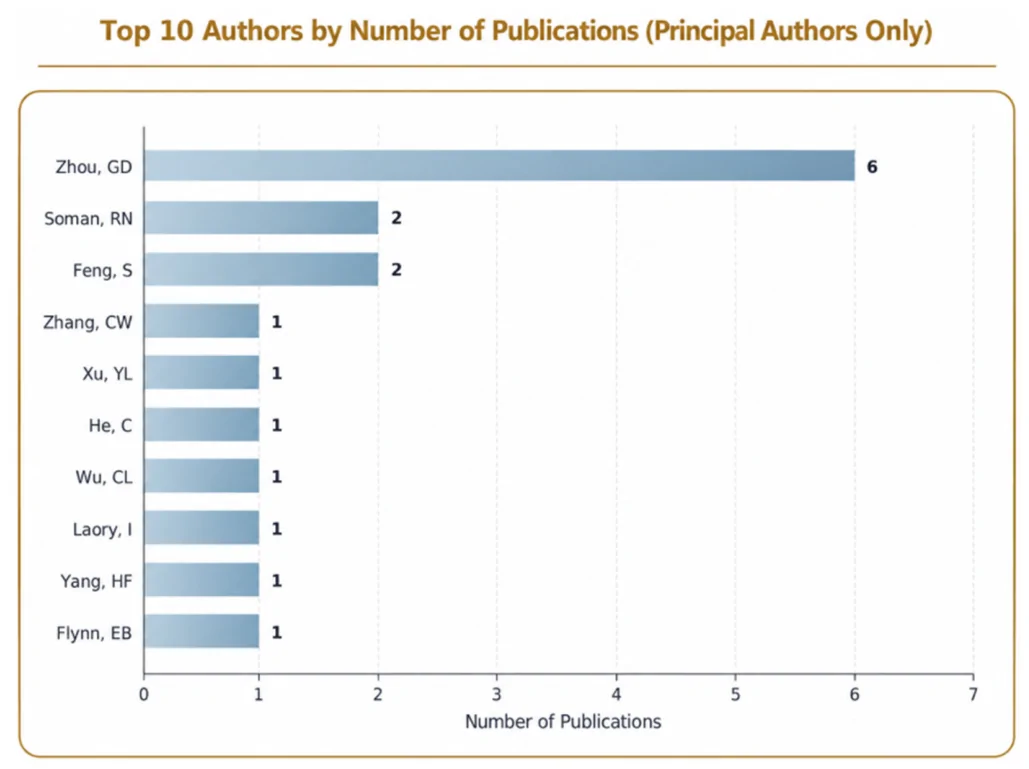
Top 10 Leading Authors by Number of Publications (Principal Authors Only).

**Figure 11:**
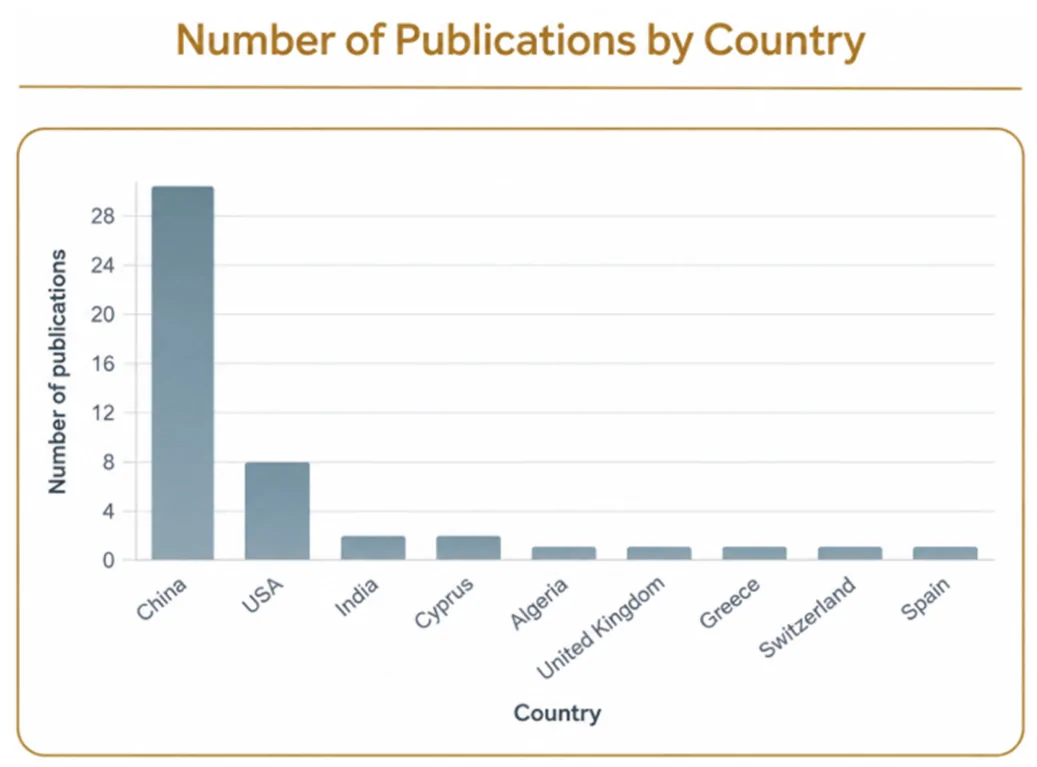
Publications reviewed by country.

**Figure 12:**
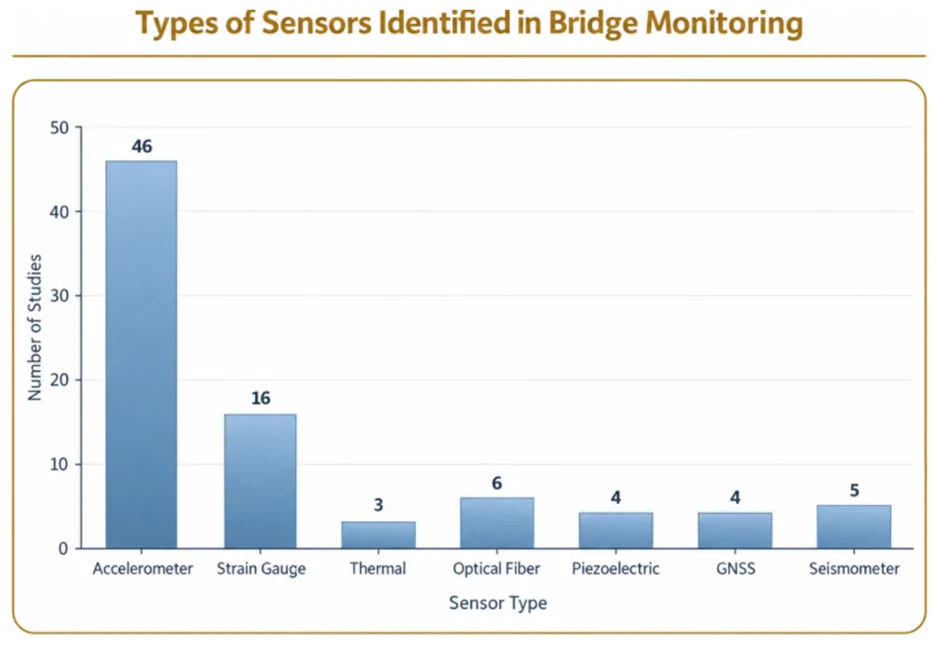
Distribution of sensor types in bridge monitoring.

**Figure 13:**
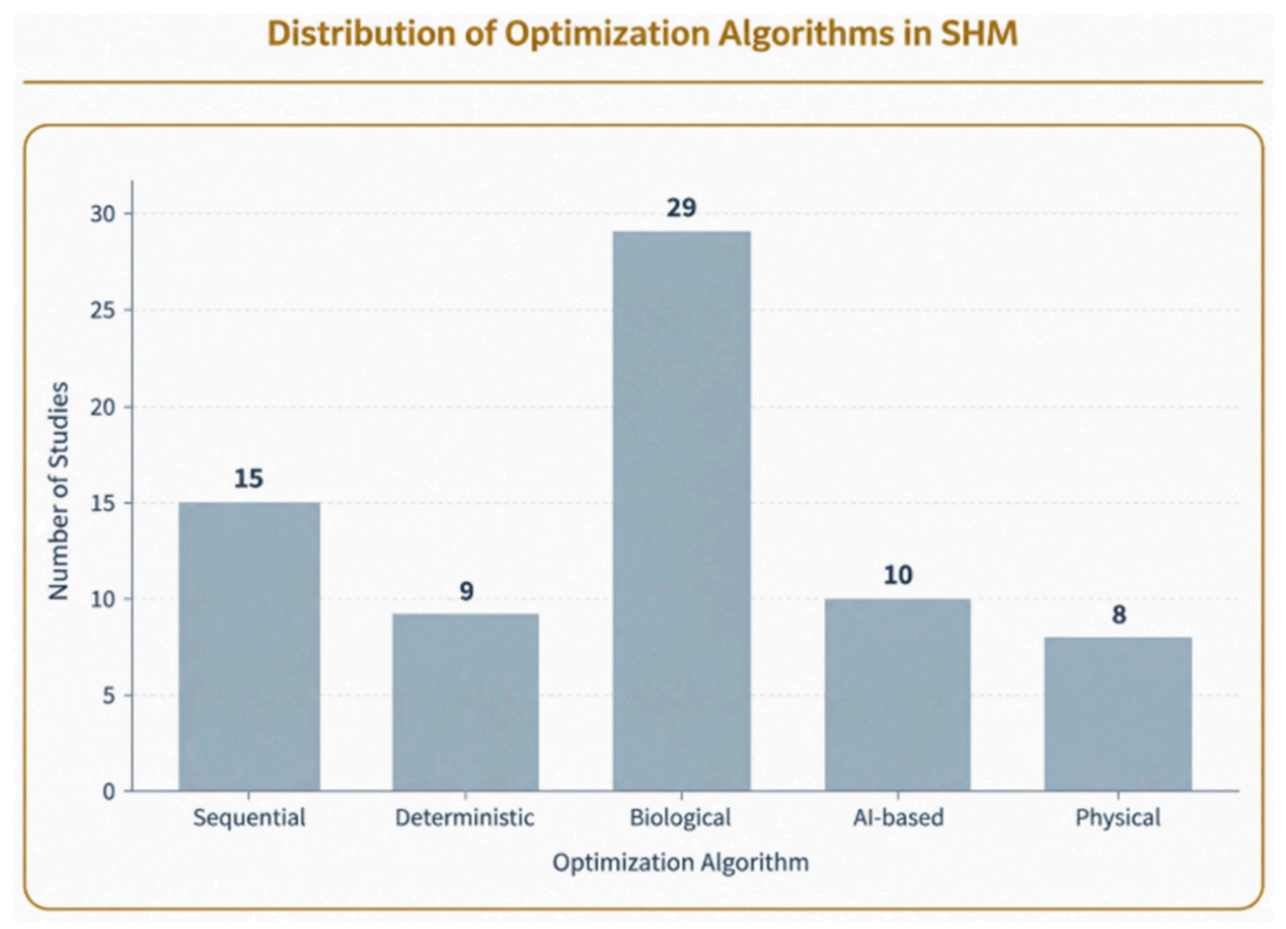
Categorization of optimization algorithms.

**Figure 14:**
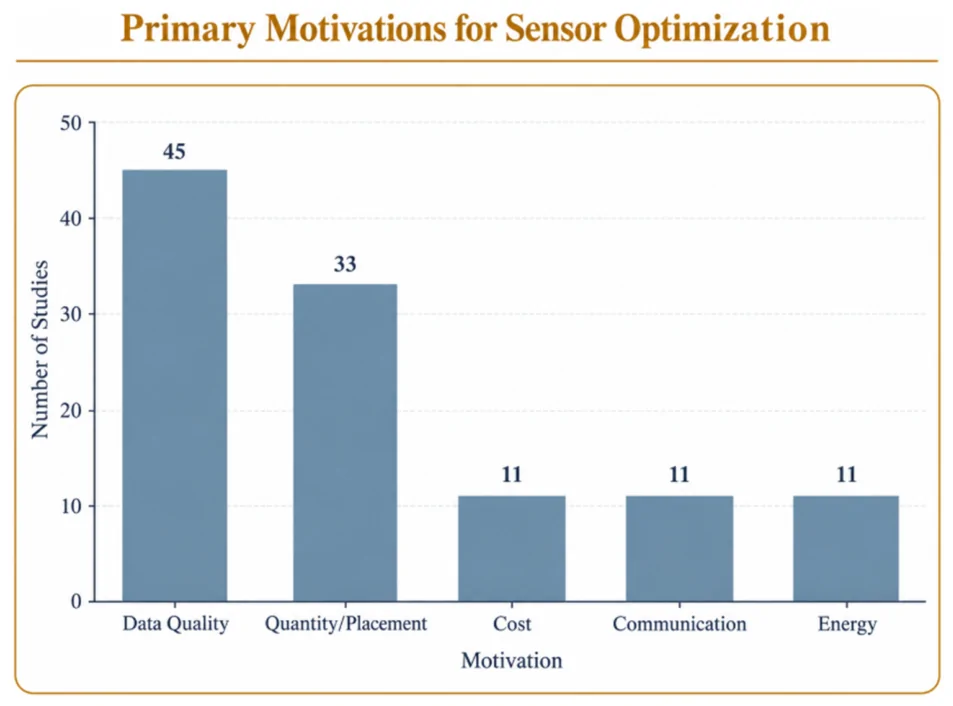
Primary motivations for sensor placement research.

**Figure 15:**
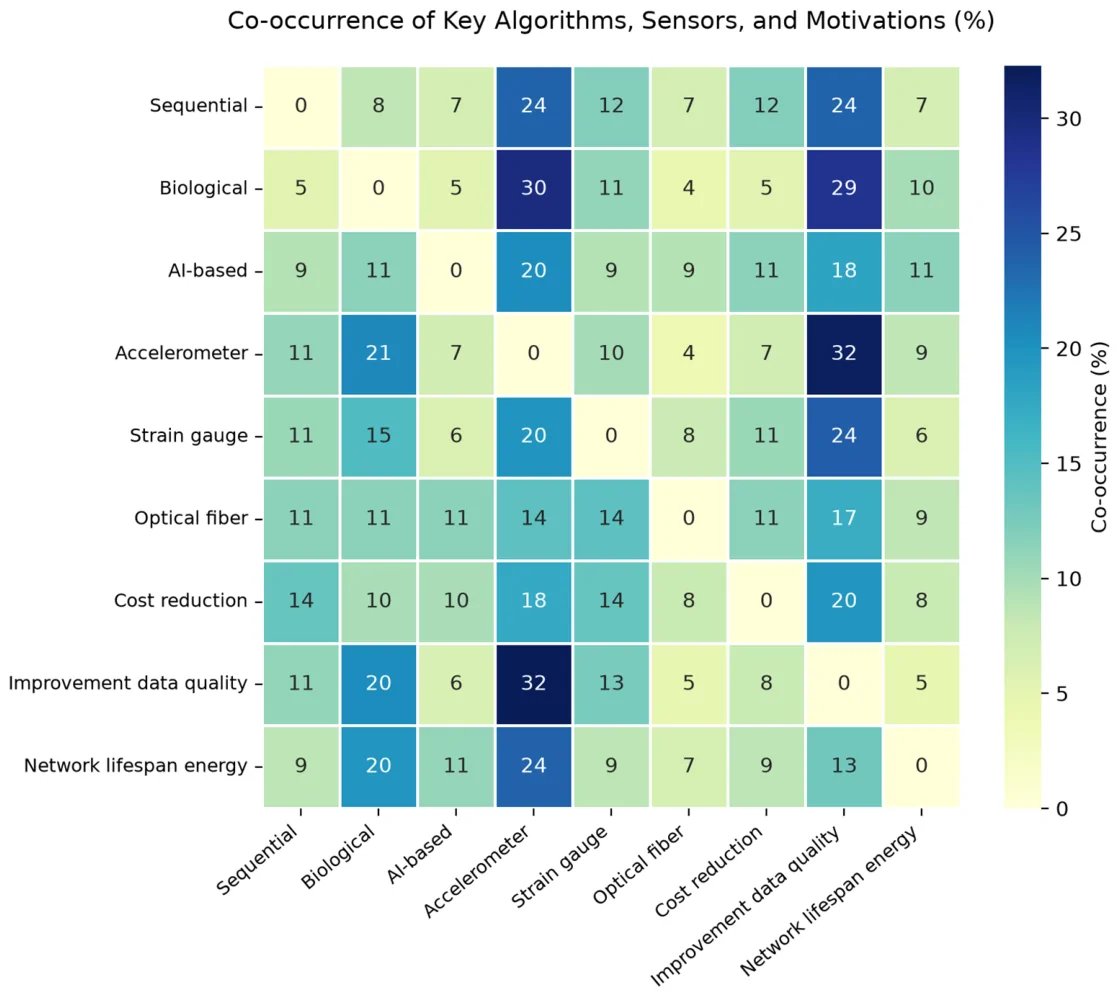
Reduced co-occurrence heatmap showing the frequency with which key optimization paradigms, sensor modalities and primary research motivations appear together in the reviewed studies. Values are expressed as percentages of the row category total.

**Figure 16:**
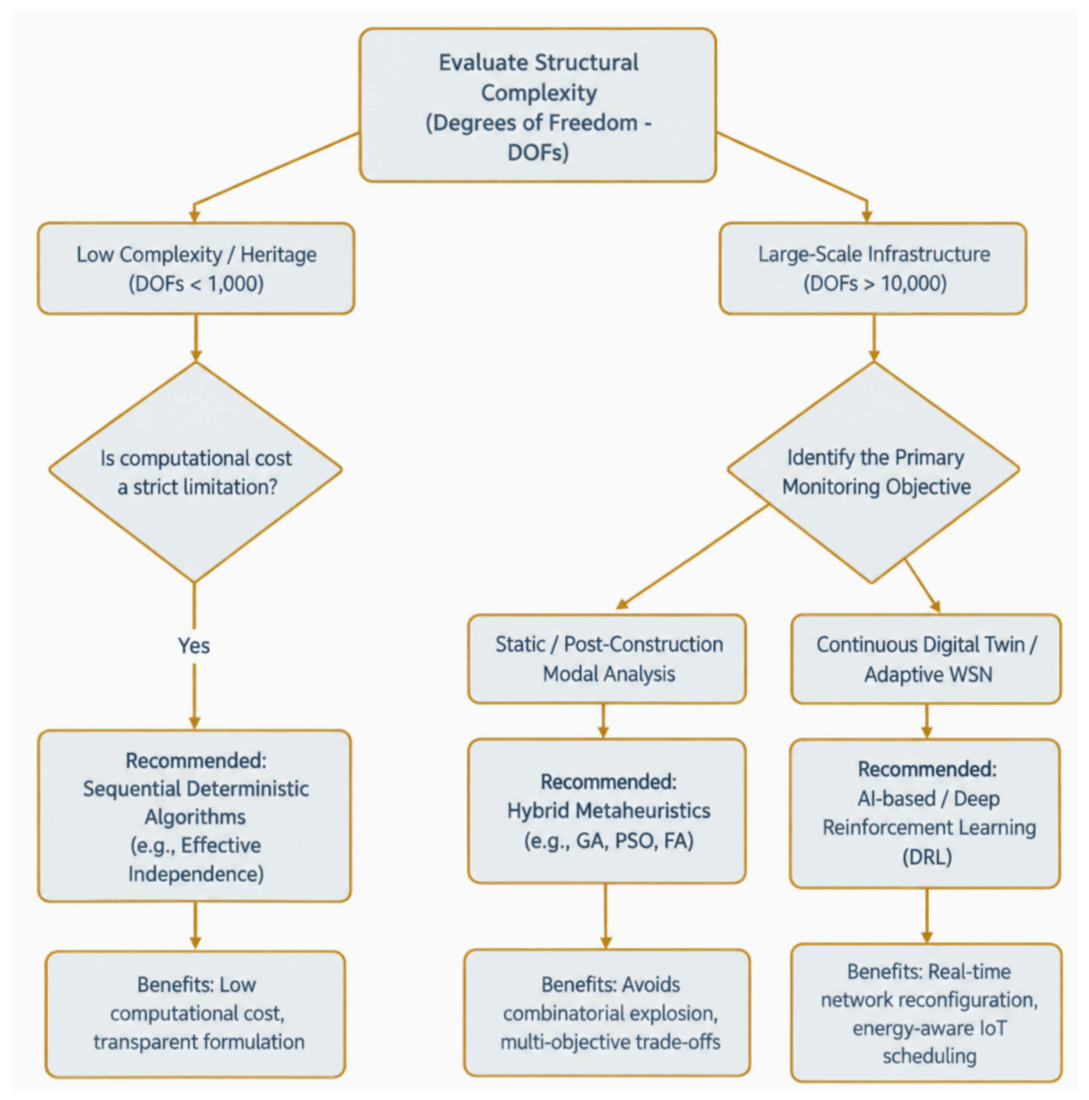
Operational Decision Tree Framework for OSP algorithm selection based on structural complexity and primary monitoring objectives.

**Table 1:** Phrases and queries used in the article search.

Database	Query
WoS	(TS=(“sensor placement” OR “sensor positioning” OR “sensor network optimization”) AND TS=(“bridge health monitoring” OR “structural health monitoring” OR “bridge monitoring”) AND (TS=(“metaheuristic” OR “optimization” OR “genetic algorithm” OR “particle swarm optimization”))) NOT TS=(“building” OR “dam” OR “tunnel”)
IEEE Xplore	(“sensor placement” OR “sensor positioning” OR “sensor network optimization”) AND (“bridge health monitoring” OR “structural health monitoring” OR “bridge monitoring”) AND (“metaheuristic” OR “optimization” OR “genetic algorithm” OR “particle swarm optimization”) NOT (“building” OR “dam” OR “tunnel”)

**Table 2:** Inclusion and exclusion criteria.

Criterion Type	Criterion	Description	No.
Inclusion	Studies on sensor placement in bridges	Includes studies specifically focused on bridges or presenting models or theories applicable to these structures. (e.g., probabilistic approaches for optimum sensor placement in bridges)	IC1
	Qualitative or quantitative analysis	Accepts studies that provide valuable insights, whether qualitative or quantitative.	IC2
	Publications from the last 15 years	Includes studies from 2010 to 2025 to capture the complete maturity cycle of metaheuristic algorithms and the consolidation of wireless intelligent monitoring systems in bridge engineering.	IC3
	Diversity in sensors and monitored variables	Includes studies utilizing a variety of sensors and monitoring different variables, such as acceleration, strain, temperature, etc.	IC4
Exclusion	Studies not focused on bridges	Excludes research focused on other structures or industries, such as aerospace Structural Health Monitoring (SHM) or healthcare data-driven algorithms, unless their models or techniques are clearly applicable to bridges.	EC1
	Lack of peer review or low quality	Excludes studies that do not meet quality standards or lack peer review.	EC2
	Studies without structural validation	Excludes studies that lack a quantitative validation phase (either through high-fidelity numerical simulations, such as Finite Element Models, or experimental tests) specifically applied to bridge structures.	EC3

**Table 3:** Reviewed articles selected for the systematic review.

No.	Authors	Year	Title	Country
1	B. Li et al. [6]	2010	High-Quality Sensor Placement for SHM Systems: Refocusing on Application Demands	China
2	B. Luo et al. [11]	2022	A Novel Harmony Search Cat Swarm Optimization Algorithm for Optimal Bridge Sensor Placement	China
3	Chai et al. [12]	2022	Optimal sensor placement of bridge structure based on sensitivity-effective independence method	China
4	Chen et al. [8]	2024	Wireless Sensor Placement Optimization for Bridge Health Monitoring: A Critical Review	China
5	Cherid et al. [13]	2022	Sensor number and placement optimization for detection and localization of damage in a suspension bridge using a hybrid ANN-PCA reduced FRF method	Algeria
6	D. Shan et al. [14]	2011	Optimal Sensor Placement for Long-span Railway Steel Truss Cable-stayed Bridge	China
7	D. Xiao et al. [15]	2022	A Strategy for Optimal Sensor Placement in Modal Signal-to-noise Ratio-based Structural Health Monitoring	China
8	Dong et al. [16]	2018	Cable force monitoring and prediction for cable group of long-span cable-supported bridges	China
9	Febrianto et al. [17]	2022	Digital twinning of self-sensing structures using the statistical finite element method	United Kingdom
10	Feng and Jia [18]	2016	3D sensor placement strategy using the full-range pheromone ant colony system	China
11	Feng and Jia [19]	2018	Acceleration sensor placement technique for vibration test in structural health monitoring using microhabitat frog-leaping algorithm	China
12	Flynn and Todd [20]	2010	An active sensor placement optimization strategy using Bayesian experimental design	USA
13	G. Li et al. [21]	2023	Sensor Optimization Placement for Bridge Structural Health Monitoring Using Improved COOT Algorithm	China
14	G. W. van der Linden et al. [22]	2011	Optimal sensor placement for health monitoring of civil structures	USA
15	Gao et al. [23]	2021	Optimal Three-Dimensional Sensor Placement for Cable-Stayed Bridge Based on Dynamic Adjustment of Attenuation Factor Gravitational Search Algorithm	China
16	Guo et al. [24]	2017	Optimal sensor placement for damage detection of bridges subject to ship collision	China
17	He et al. [25]	2013	A Combined Optimal Sensor Placement Strategy for the Structural Health Monitoring of Bridge Structures	China
18	J. C. Avendano et al. [26]	2021	Optimization of Sensor Placement in a Bridge Structural Health Monitoring System	USA
19	J. Yang and Z. Peng [27]	2020	Beetle-Swarm Evolution Competitive Algorithm for Bridge Sensor Optimal Placement in SHM	China
20	Karakostas et al. [9]	2024	Seismic assessment of bridges through structural health monitoring: a state-of-the-art review	Greece
21	Laory et al. [7]	2012	Measurement System Configuration for Damage Identification of Continuously Monitored Structures	Switzerland
22	Li et al. [28]	2020	New Sensor Placement Method Based on Remodeling Innovation	China
23	Li et al. [29]	2015	Optimal sensor placement for long-span cable-stayed bridge using a novel particle swarm optimization algorithm	China
24	Li et al. [30]	2020	Dynamic deflection monitoring of high-speed railway bridges with the optimal inclinometer sensor placement	China
25	Liu et al. [31]	2021	A two-stage optimal sensor placement method for multi-type structural response reconstruction	China
26	M. R. Banik and T. Das [32]	2019	An Integer-Constrained Genetic Algorithm for Sensor Placement in Structural Health Monitoring	India
27	Martin and Droguett [1]	2024	Quantum-Based Combinatorial Optimization for Optimal Sensor Placement in Civil Structures	USA
28	Meng et al. [5]	2023	Optimal sensor placement for structural health monitoring based on deep reinforcement learning	China
29	Pachón et al. [33]	2018	E. Torroja’s bridge: Tailored experimental setup for SHM of a historical bridge with a reduced number of sensors	Spain
30	S et al. [34]	2024	Multiple criteria-based sensor optimization for Structural Health Monitoring of civil engineering structures	India
31	Soman et al. [35]	2014	Multi-type, multi-sensor placement optimization for structural health monitoring of long span bridges	Cyprus
32	Soman et al. [36]	2012	Optimization of Wireless Sensor Locations for SHM based on application demands and networking limitations	Cyprus
33	van der Linden et al. [37]	2010	Near-Optimal Sensor Placement for Health Monitoring of Civil Structures	USA
34	Wang et al. [10]	2023	Advancements in Optimal Sensor Placement for Enhanced Structural Health Monitoring: Current Insights and Future Prospects	China
35	Wu et al. [4]	2013	Sensor Optimal Placement for Structural Health Monitoring based on Stabilization Diagram	China
36	Wu et al. [38]	2019	Development of sensor placement optimization tool and application to large-span cable-stayed bridge	USA
37	Xu et al. [39]	2016	Multi-type sensor placement and response reconstruction for structural health monitoring of long-span suspension bridges	China
38	Y. Zhang et al. [40]	2022	Reinforcement learning-based IoT sensor scheduling strategy for bridge structure health monitoring	China
39	Yang et al. [41]	2012	Research on Optimal Sensor Placement Based on Reverberation Matrix for Structural Health Monitoring	China
40	Yang et al. [42]	2024	Structural Health Monitoring of Long-Span Continuous Girder Bridge: System Implementation and Data Analysis	China
41	Zhang et al. [43]	2025	Optimal sensor placement and structural health monitoring methods of ancient stone bridges based on an improved genetic algorithm: Taking Lugou Bridge as an example	China
42	Zhang et al. [44]	2016	OPTIMAL SENSOR PLACEMENT USING CHAOTIC MONKEY SEARCH ALGORITHM	USA
43	Zhou et al. [45]	2019	Optimal wireless sensor network configuration for structural monitoring using automatic-learning firefly algorithm	China
44	Zhou et al. [46]	2014	Wireless Sensor Placement for Bridge Health Monitoring Using a Generalized Genetic Algorithm	China
45	Zhou et al. [47]	2017	Wireless sensor placement for structural monitoring using information-fusing firefly algorithm	China
46	Zhou et al. [2]	2015	A Comparative Study of Genetic and Firefly Algorithms for Sensor Placement in Structural Health Monitoring	China
47	Zhou et al. [48]	2015	Energy-aware wireless sensor placement in structural health monitoring using hybrid discrete firefly algorithm	China
48	Zhou et al. [49]	2015	Optimal sensor placement under uncertainties using a nondirective movement glowworm swarm optimization algorithm	China
49	Zhou and Wu [50]	2017	Strain gauge placement optimization for structural performance assessment	USA

**Table 4:** Quality Assessment and Risk of Bias scoring rubric.

Score	Validation Method	Structural Complexity and Scalability	Reproducibility and Transparency	Robustness Against Uncertainty
3	Experimental validation on a physical bridge structure or full-scale field deployment.	Applied to large-scale structures with thousands of degrees of freedom (DOFs ≥ 10,000).	Explicit formulation of the objective function, full hyperparameter disclosure, and open-source code.	Quantitative robustness evaluation considering multiple realistic sources of uncertainty, such as measurement noise, environmental variability, or sensor failure.
2	High-fidelity numerical simulation utilizing complex 3D Finite Element Models (FEM).	Applied to medium-scale structures (1000 ≤ DOFs < 10,000).	Clear mathematical formulation and parameter disclosure, but no source code provided.	Quantitative robustness evaluation considering at least one source of uncertainty or measurement noise.
1	Basic numerical validation on simplified 2D structures or theoretical beam models.	Applied to small-scale or highly reduced models (DOFs < 1000).	Partial description of the objective function or missing critical hyperparameters.	Uncertainty is acknowledged or qualitatively discussed, but no quantitative robustness assessment is performed.
0	Absence of explicit structural validation.	Scalability limitations explicitly restrict practical application.	Insufficient methodological detail to replicate the optimization.	No explicit consideration of uncertainty, noise measurement, environmental variability, or sensor failure.

**Table 7:** Unified Decision Matrix for OSP Algorithm Selection.

\	Scalability (DOFs)	Optimality Guarantee	Best For (Bridge Type)	Computational Cost	Viability (Civil Gap)	Recommended Engineering Scenario	Technical Constraints
Sequential (EFI)	Limited; most effective for reduced models or historical structures with <10^3^ DOFs.	Local optimum; selection is irreversible due to greedy nature.	Simple beam/arch bridges or historical heritage structures.	Very Low; step-by-step linear algebra evaluations.	High; preferred by civil engineers for its simplicity.	Preliminary Monitoring/Heritage Structures.	Strictly limited budget; preserve historical integrity.
Metaheuristic (GA/PSO)	High; capable of exploring massive search spaces in long-span bridges.	Near-global; balances exploration and exploitation.	Complex long-span, railway steel truss, or cable-stayed bridges.	Moderate; iterative procedures and convergence tuning.	Medium; requires specialized knowledge in metaheuristic tuning.	Long-Span Bridges (Cable-stayed/Suspension).	Multiple coupled modes; massive combinatorial search spaces.
AI-Based (DRL/ML)	Very High; manages high-dimensional big data and IoT environments.	Probabilistic; performance learned through reward mapping.	Smart structures requiring IoT scheduling or adaptive WSN.	High training/Low inference; real-time capable post-deployment.	Critical; needs deep collaboration between civil and CS experts.	Digital Twins/Real-Time SHM.	High data dimensionality; continuous virtual sensing required.
Deterministic (Analytical)	Low; limited by NP-hard nature of large-scale bridge models.	Absolute; exact mathematical proofs for well-posed problems.	Simplified theoretical models or simply supported beams.	Extremely High for structures with thousands of DOFs.	High in theory; low in practical field implementation.	Academic/Reference Models (small-scale validation).	Simplifying assumptions required; computationally prohibitive for real bridges.
Hybrid (Firefly, Multi-Objective)	Medium to High; excels in multi-objective formulations.	Multi-objective; balances energy, coverage, and data quality.	WSN deployments on medium to large bridges.	Moderate to High; multi-objective adds computational overhead.	Medium; emerging with demonstrated WSN efficiency.	Wireless Sensor Networks (WSN) Deployment.	Critical battery consumption; data routing and connectivity coverage.

**Table 8:** Qualitative comparison and typical conditions of use of the main SHM sensing modalities.

Sensor Modality	Primary Application	Relative Cost/Scalability	Diagnostic Sensitivity	Typical Condition of Use
Accelerometers	Global modal analysis (frequencies, mode shapes)	Low/High	High for global stiffness loss; Low for micro-cracks	Global monitoring of long-span bridges; placement at modal anti-nodes.
Strain Gauges	Local deformation and stress monitoring	Low/Moderate	High for localized material yielding	Critical load-bearing elements; fatigue-prone welded joints.
Optical Fiber (FBG)	High-density strain and temperature sensing	High/Moderate	Very High; immune to electromagnetic interference	Environments with high thermal variation or electromagnetic noise.
GNSS	Absolute 3D static/dynamic displacement	Very High/Low	Moderate; limited to macro-displacements	Real-time monitoring of pylon sway and mid-span deflection in extreme weather.
Piezoelectric	High-frequency acoustic emission/micro-cracks	Moderate/Moderate	Very High for early-stage crack propagation	Direct integration into concrete or steel to monitor active crack growth.

## Data Availability

The data presented in this study are available within the article (see Table 3 and Table 5).
